# Male and female contributions to diversity among birdwing butterfly images

**DOI:** 10.1038/s42003-024-06376-2

**Published:** 2024-07-01

**Authors:** Jennifer F. Hoyal Cuthill, Nicholas Guttenberg, Blanca Huertas

**Affiliations:** 1 School of Life Sciences, University of Essex, Colchester, UK; 2 Cross Labs, Cross Compass Ltd, Tokyo, Japan; 3 Department of Science, Natural History Museum, London, UK

**Keywords:** Sexual selection, Machine learning, Phylogenetics

## Abstract

Machine learning (ML) newly enables tests for higher inter-species diversity in visible phenotype (disparity) among males versus females, predictions made from Darwinian sexual selection versus Wallacean natural selection, respectively. Here, we use ML to quantify variation across a sample of > 16,000 dorsal and ventral photographs of the sexually dimorphic birdwing butterflies (Lepidoptera: Papilionidae). Validation of image embedding distances, learnt by a triplet-trained, deep convolutional neural network, shows ML can be used for automated reconstruction of phenotypic evolution achieving measures of phylogenetic congruence to genetic species trees within a range sampled among genetic trees themselves. Quantification of sexual disparity difference (male versus female embedding distance), shows sexually and phylogenetically variable inter-species disparity. *Ornithoptera* exemplify high embedded male image disparity, diversification of selective optima in fitted multi-peak OU models and accelerated divergence, with cases of extreme divergence in allopatry and sympatry. However, genus *Troides* shows inverted patterns, including comparatively static male embedded phenotype, and higher female than male disparity – though within an inferred selective regime common to these females. Birdwing shapes and colour patterns that are most phenotypically distinctive in ML similarity are generally those of males. However, either sex can contribute majoritively to observed phenotypic diversity among species.

## Abstract (web summary)

Machine learning on over 16 thousand photos of birdwing butterflies detected both examples of higher male than female variation, and vice versa, testing predictions of natural versus sexual selection debated by Darwin and Wallace.

## Introduction

Two opposed hypotheses for the evolution of sexually variable animal phenotypes are sexual selection on phenotypes of the sex chosen for mating, proposed by Darwin^1–4^, or natural selection on the sex with greater reproductive input, suggested by Wallace^5,6^. Birdwing butterflies include spectacular examples of sexually dimorphic colour, pattern, and shape^7^. Within the swallowtail butterfly family Papilionidae, the birdwing butterflies present a morphological and biogeographic diversification, across the Pacific region, into three recognised genera, *Trogonoptera* Rippon, 1890, *Troides* Hübner, 1819 and *Ornithoptera* Boisduval, 1832^8,9^, with 37 species^8^ and >130 subspecies^10^. Often rare, dependent on tropical forest^11,12^, and long-prized by collectors for their exceptional beauty^7^ and size, all birdwing species are under legal protection for trading by CITES, and some are categorised as endangered species^8^. Morphological studies have shown divergence in both wing colouration mechanisms and photoreceptor excitation between examples of birdwing butterfly species, suggesting that wing morphology may function in mate recognition and signalling^13–16^. Qualitative observations have suggested, first, that male birdwings can be more brightly coloured than females^17^, and, second, that males from different islands can, in cases, differ more markedly from each other than do corresponding females (e.g., *Ornithoptera*, Solomon Islands^18^). Furthermore, birdwing butterflies exhibit elaborate mating behaviour, including male courtship displays subject to female mate choice^19^ (SI Supplementary Note 1). Birdwing males have been observed to pursue multiple females, whereas females have been observed to reject matings^12^, implying a steeper relationship between numbers of mates and direct reproductive success for males than females^20^. Such asymmetries theoretically predict sexual selection on phenotypes of males from mating preferences of females^2,21^. This suggests the hypothesis that sexual selection, acting on phenotypes primarily of males, first proposed by Darwin^1^, has been correlated with, and potentially causative of, both sexual dimorphism and inter-species phenotypic diversification^22–25^. Notably, however, female evolution may also make distinct contributions to phenotypic variation, under a less studied^6^ hypothesis originally presented by Wallace^5^. Wallace predicted that females under comparatively high natural selection pressure for protective phenotypes, due potentially, for example, to higher predation rates^5,6,26^, or lower sexual selection on female phenotype (making natural selection relatively more important), will show divergent allopatric wing patterns, in response to habitat variation^5^.

Here we capture an unprecedented extent of phenotypic variation among both birdwing females and males to address this historical imbalance and reveal the extent of female variation. Sexually dimorphic groups in which females are morphologically more diverse than males have only rarely been identified^3,6,27,28^. The effects of sexual dimorphism on inter-species diversity of phenotypes (i.e., disparity: the multidimensional extent of variation in phenotypes^29,30^) have been tested relatively rarely using any methods^31,32^ and new capacities for quantification among comprehensive samples of whole-organism photographs are only recently enabled by new methods of machine learning (Figs. S1–S3). Furthermore, though pivotal studies have shown that it is possible to compare samples of female and male disparity, using traditional morphometric methods, it has remained common practice in studies of sexual dimorphic biological traits to focus on male traits^31^. Consequently, machine learning methods open up new possibilities to quantify the extent to which male and female variation, predicted by sexual and natural selection^1,5^, contributes to the diversity of large-scale photographic samples of overall visible phenotype.

Further to this, biological signalling may, in general, be under multiple, potentially conflicting evolutionary selection pressures, selection strengths (including potential neutrality), and constraints, presenting a range of alternative causes for the evolution of biological signals. Among birdwing butterflies, as well as other animal groups, potential selection pressures on visible phenotype include species recognition^5^, sexual selection, predation, potentially effecting aposematic warning colouration (given the toxicity of birdwing larval hostplants^33^), and/or camouflage (across potentially variable viewing distances and backgrounds), and flight^19^, with potential for sexual variation in natural selection on males, as well as females^34^.

Here, we use machine-learnt embeddings to quantify and characterise, relative to predictions of sexual versus natural selection in phenotypic diversification^1–6^, sexual and interspecific variation across 16,734 dorsal and ventral photographs of birdwing butterflies, covering the entire Natural History Museum (NHMUK) birdwing collection, the largest and most comprehensive known on this group, including the three genera, 35 species OTUs (operational taxonomic units) and 131 recognised subspecies. Until very recently, methods capable of quantitatively capturing phenotypic variation approaching this scale and complexity did not exist^30,35–42^. We use deep learning with a triplet-trained convolutional neural network (CNN), ButterflyNet version 1.2 (Supplementary Software 1, modified from ButterflyNet version 1^35^), to generate Euclidean spatial embeddings of uniformly scaled, dorsal and ventral photographs. Supervised CNNs can learn visually similar image features directly from biological images that are labelled, for example, by species^35,39,40^. Embedding methods based on such CNNs can then capture the extent and relational structure of image similarity within multidimensional image embeddings^35,39,43,44^, comparable to biological “morphospaces” constructed using traditional morphometric methods^30^. Because CNNs are comparatively robust to translation, rotation, and scaling, can compare one-to-many features, and can access directly any image variation informative for their task (without subjective variable selection), their capabilities extend beyond previous methods of biological image comparison, such as geometric morphometrics or pixel colour comparison^35,39,40,45^ (Figs. S1–S3).

Here, our applications of ML to whole-specimen photographs facilitate the first: (i) Tests of congruence between learnt phenotypic distances and genetic phylogenetic distances, relative to those among genomic data, with ML training time; relevant to ML validation and its potential for automated morphological phylogenetic reconstruction. (ii) Quantitative morphometric analysis of the sexually dimorphic birdwing butterflies (*Trogonoptera*, *Troides,* and *Ornithoptera*), a case study for the extent of sexual and inter-species variation in phenotype. (iii) Tests, among whole-specimen photographs, of male versus female contributions to observed inter-species variation (disparity) hypothesised under sexual^1^ versus natural^5^ selection. (iv) Applications of phylogenetic comparative measures to infer selective optima and diversification rates among phenotypic metrics learned directly by ML triplet embedding. (v) Comparisons of machine-learnt phenotypic distinctiveness, in allopatry and sympatry.

## Results and discussion

### Machine-learnt triplet embeddings

The 16,734 images of birdwing butterfly specimens were embedded in a Euclidean multidimensional space capturing the visible similarity informative to a CNN (Supplementary Software 1). The network architecture (v. 1.2) builds on ButterflyNet 1 to incorporate the following key features: optimising image embedding using triplet loss^35,36,43,44^ (without an additional classification loss^35^), adding image augmentation and calculating “live” correlations with genetic distance, visualised during training, that facilitate comparisons of embedding distances to independent genetic distances. The CNN was trained to optimally place all images (sampled in triplets, two of the same species, one different) such that the Euclidean distances between images from the same species are comparatively close, relative to distances to images of different species.

**Fig. 1:**
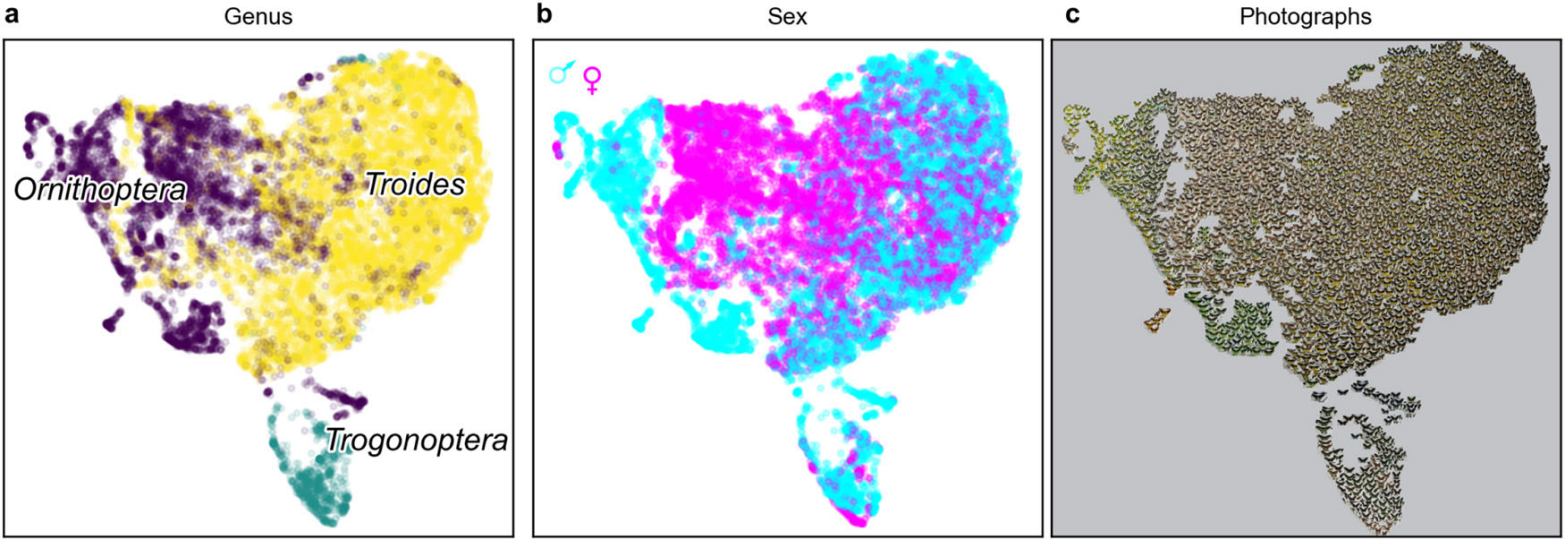
Patterns of phenotypic similarity in birdwing butterfly genera and sexes. Early structure in a machine-learnt embedding of 16,734 dorsal and ventral birdwing butterfly photographs, trained for 10 epochs on triplets of photographs with species OTU labels. Points represent individual photographs. Embedded proximity represents image similarity. Visualisations of a 64-dimensional ML embedding, projected to 2D using the UMAP^46^ algorithm. Structure of genera (**a**), biological sexes (**b**), and the embedded images (**c**).

Visualisations of the resultant embeddings highlight biologically meaningful structure (Fig. 1), using projection to 2D via the UMAP^46^ algorithm. UMAP (Uniform Manifold Approximation and Projection) aids interpretation of structure, such as clustering, in multidimensional spaces (here our 64-dimensional machine-learnt embedding) by using geometric and topological methods^46^ to project the data to a lower number of dimensions (here 2) that can be more easily visualised.

**Fig. 2:**
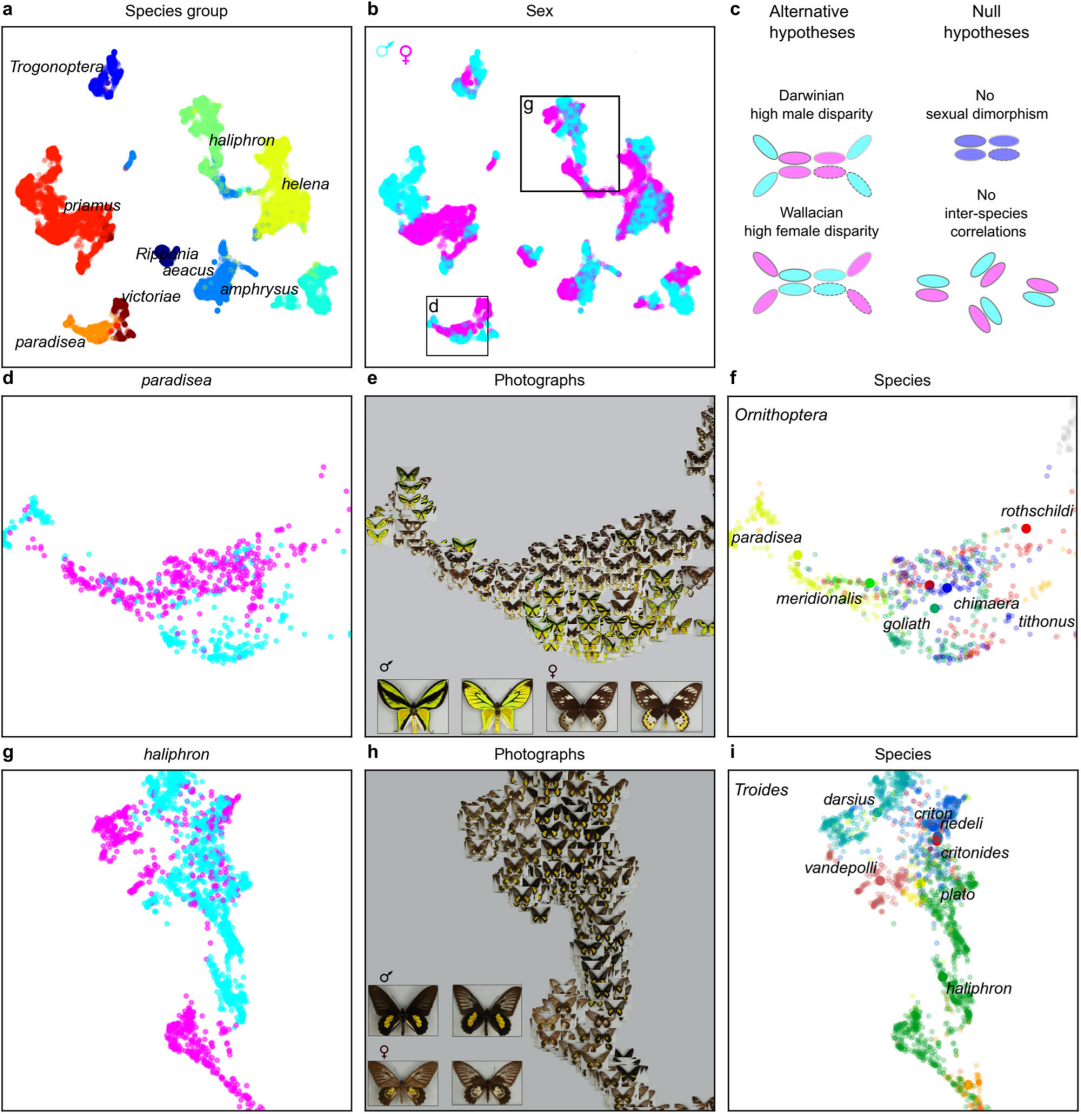
Patterns of phenotypic similarity in birdwing species groups and sexes. Late structure of machine-learnt embedding trained for 2990 epochs (*n* = 16,734 images, as for Fig. 1). Structure of species groups (**a**) and sexes (**b**). **c** Diagram illustrating alternative and null evolutionary hypotheses (clusters for each species represented by ovals sharing border colour). **d**–**i**, Exemplar species groups showing inverted patterns of sexual disparity (**d**, **g**, sexes; **e**, **h**, photographs; **f**, **i**, species). **d**–**f** *Ornithoptera paradisea* species group showing clustered females with peripheral males (neighbour-joining clade depth females 0.32, males 0.53). **g**–**i** *Troides haliphron* species group showing clustered males with peripheral females (clade depth females 1.0, males 0.31). Large inset photographs show examples of male and female, dorsal (left) and ventral (right) for *O. paradisea* (**e**) and *T. haliphron* (**h**).

**Fig. 3:**
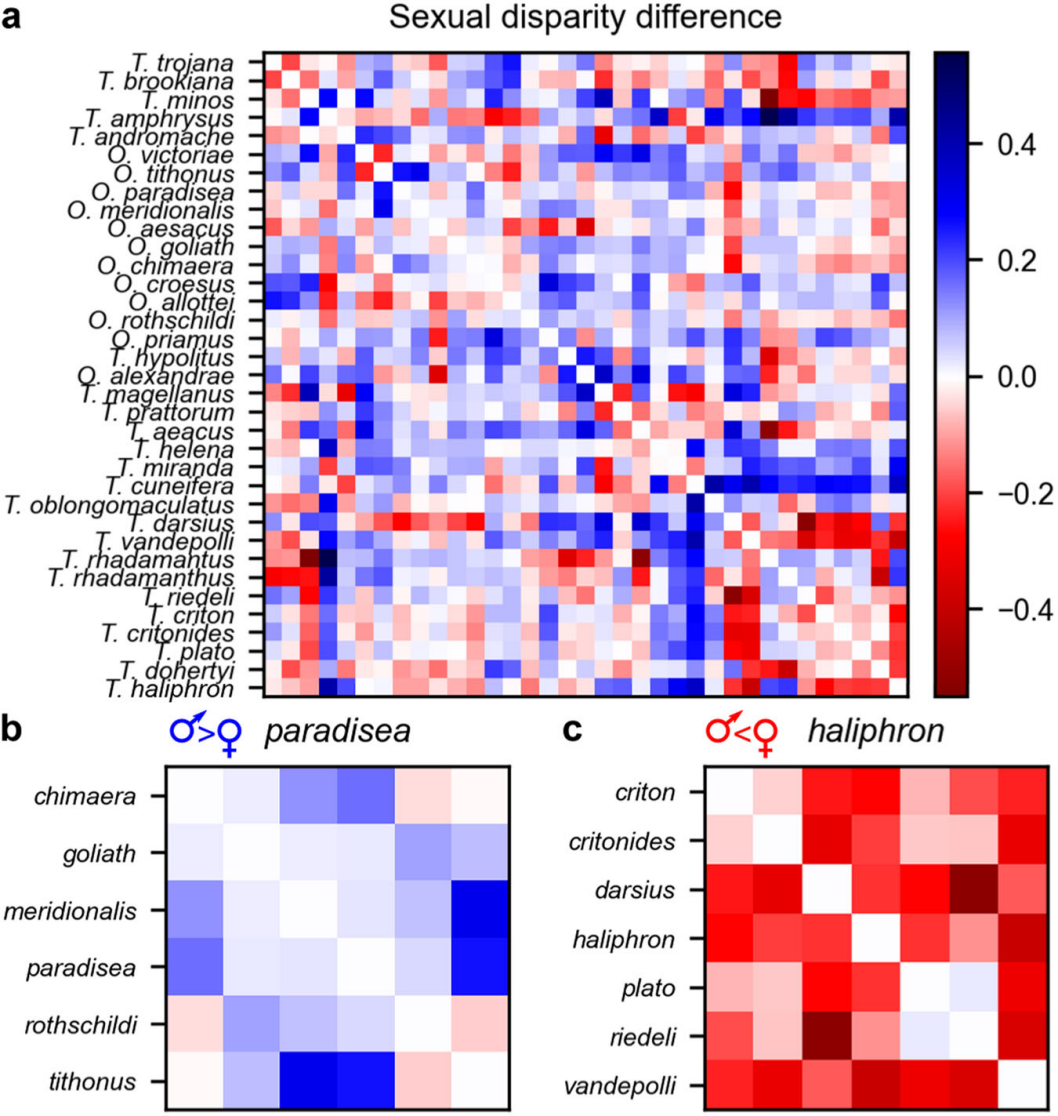
Sexual disparity among birdwing butterfly species. **a**–**c** Heatmaps showing sexual disparity difference: the difference between pairwise inter-species distances for males versus corresponding females, based on an ML phenotypic embedding trained for 2990 epochs (*n* = 16,734 images, as for Fig. 1). When the difference is positive (blue), males are more disparate than females. When the difference is negative (red), females are more disparate than males. **a** All 35 included species OTUs (phylogenetic tip order). **b**, **c** Exemplar species groups showing polarised average sexual disparity (alphabetic order). **b** *Ornithoptera paradisea* species group (Fig. 2d–f), positive mean = 0.06. **c** *Troides haliphron* group (Fig. 2g–i), negative mean = −0.19). Column order, left to right, repeats row order, top to bottom.

**Fig. 4:**
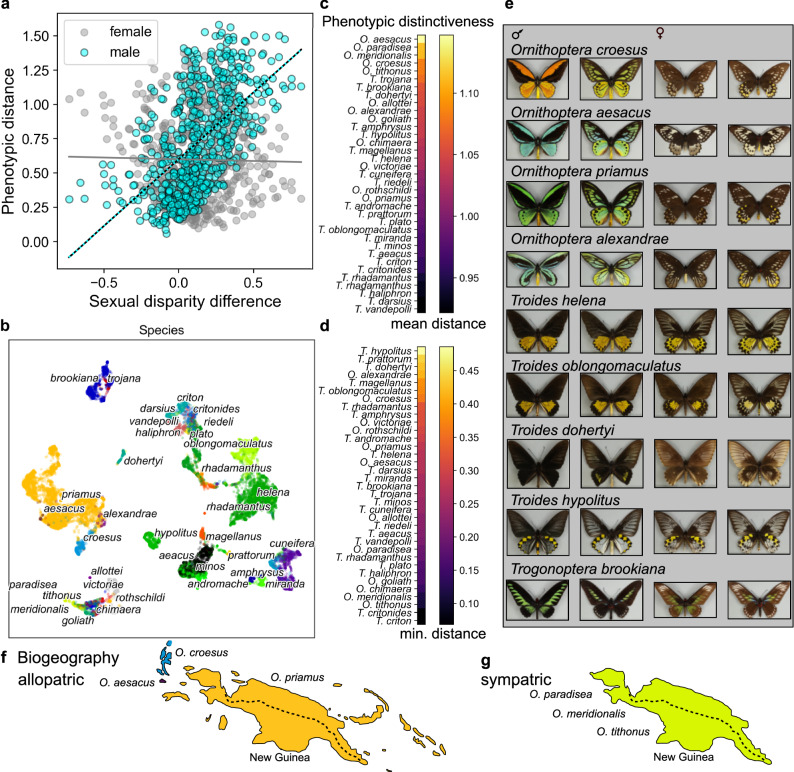
Phenotypic distinctiveness and sexual disparity. **a** Relationships between centroid phenotypic distances between males or females of birdwing species and inter-species sexual disparity difference (positive difference: inter-male distance > inter-female distance; negative difference: inter-female distance > inter-male distance). Early embedding trained for 10 epochs (corresponding to Fig. 1). **b** Late machine-learnt phenotypic embedding trained for 2990 epochs, image points coloured by species OTUs (dataset as for Fig. 1). **c**, **d** Ranked phenotypic distinctiveness of birdwing species measured by mean (**c**) or minimum (**d**) distance (ordered most distinctive, top, to least, bottom,). Averages across 15 embeddings, epoch range 10–2990, three independent training runs. **e** Illustrated examples (bold text panels **b**–**g**) of phenotypically distinctive species, left to right: male dorsal, ventral; female dorsal, ventral. See also Fig. 1, **e**. *n* = 16,734 images, as for Fig. 1. **f**, **g** Maps contrasting cases of allopatric (**f**) and sympatric (**g**) geographic ranges^12^ in phenotypically distinctive species, from independently supported^9^ species clades (Fig. S10), on New Guinea (dashed line, mountain range) and nearby islands. **f** Allopatric ranges of *O. croesus*, *O. aesacus* and *O. priamus* (also extending into Australia). **g** Sympatric ranges^12^ occur in *O. paradisea* (Fig. 2e), O*. meridionalis* and *O. tithonus* on New Guinea.

Notably, the late embedding structure includes both clusters of images with the same species identity (e.g., Fig. 2f, i), corresponding to the labels on which the algorithm was trained (Figs. 1–4, species OTU), and additional structure within and above the species level, beyond the label information to which the algorithm was given direct access (also present in earlier embeddings; Figs. 1–2, Fig. S4). Such structure can be expected to be recovered by an ML embedding because it aids success in the training task itself (in this case, placement of image triplets by species). In particular, embedding structure is present, which can be mapped to biological sex (Figs. 1b, 2b) and higher level phylogeny and taxonomy^8,11^, including genus (Fig. 1a, c) and species group (Fig. 2a).

### Validation of evolutionary embedding structure

The extent to which our machine-learnt embedding distances, based on image similarity, were correlated with independent evolutionary phylogenetic information was quantitatively validated^35^ against genetic phylogenetic signals (with phylogenetic signal defined as the information on relatedness a given measure of similarity contains, *sensu*^47^), based on published sequences from four housekeeping genes available for 177 specimens^8^, representing 30 of our 35 phenotypic species OTUs (Accession numbers Supplementary Data 2; Supplementary Software 2). These analyses compared the congruence between ML phenotypic and multi-gene coalescent species trees to a statistical distribution of genetic species tree similarities, which provides an independent reference for the extent of congruence expected between estimates of species phylogeny (given natural processes that can generate phylogenetic signal variation, conflict, and uncertainty). Measures of tree similarity between a phenotypic neighbour-joining species phylogeny were reconstructed from the ML image embedding, and 1000 multi-species coalescent genetic trees (taken from post-burn-in Bayesian MCMC chains that reflect the likelihood of sampled trees by the relative amounts of search time spent in different regions of tree space). These comparisons show that the distribution of distances between machine-learnt phenotypic trees and independent genetic trees overlaps that among genetic species trees themselves, with a median within their internal range (Fig. S5). Distributions of similarity between ML phenotypic trees and genetic trees were also found to be very significantly different from those expected at random (*n* = 1000, Kruskal–Wallis, two-tailed *p* < 0.001, Fig. S6).

### Embedding structure and training time

During ML training, triplet loss (which the algorithm aims to minimise) successfully tends to converge towards a stable level (Supplementary Software 1; Fig. S7). Applications of embedding methods, which pass a hurdle of successful convergence in their training loss function, then present new questions of the extent of correlation between the measures of image similarity recovered throughout this training process (as it approaches and achieves convergence in training loss) and further variables of biological interest. Here, in particular, we explore the correlation between embedded image similarity and independent evolutionary measures of genetic distance, as ML training proceeds. Initially, there is also a rapid increase in the correlation between inter-species genetic distances and the phenotypic embedding distances which have been learnt (Fig. S7). However, as ML training proceeds, different levels of taxonomic, phylogenetic, and phenotypic structure are revealed (Figs. 1–2, Fig. S4). For example, 2D visualisations of relatively early embedding structure (soon after initial, rapid improvement in triplet loss) indicate large clusters mapping broadly to genus and sex (Fig. 1, Fig. S4). In relatively later training, different species groups become increasingly distinct (Fig. 2). However, the deeper relationships that were marked in early embeddings can become less apparent. In line with this, in the reported training runs, the highest correlation between phenotypic embedding and genetic distances is achieved by approximately epoch 500, after which this correlation broadly declined, while triplet loss approached stabilisation around epoch 3000 (with fluctuations between individual epochs throughout) (Fig. S7). This is notable as an example of a non-linear relationship between ML optimisation (minimisation of species triplet loss) and its secondary outcome, embedding structure (particularly, evolutionary phylogenetic signal present in embedded distances), which may (as here) be an ultimate aim in using machine learning. We show here that there is not necessarily one correct stopping time, since different stopping times can capture different, biologically relevant information. Consequently, we suggest comparing results stopped at different points, particularly after rapid early improvement and later convergence in triplet loss (a procedure that can be performed whether or not independent validation data is available for a given study). The following evolutionary results compare ML phenotypic distances from different training epochs (including early stopping times soon after initial loss and genetic distance improvement as well as late stopping times at loss convergence), present averages calculated across different runs and stopping times, and meet the additional criteria that they show emergent biological structure, mapping to sex and taxonomic group, median similarity between phenotypic and genetic trees within the internal range of gene-tree similarity, and reveal evolutionary patterns consistent across independent training runs (as specified below).

### Sexual variation in visual similarity

Quantification of the extent of sexual variation in machine-learn phenotypic similarity, using embedding visualisation and a measure (calculated from embedding distances) that we call the sexual disparity difference (male minus female disparity), shows that observed inter-species disparity among birdwing butterflies is variable between biological sexes and phylogenetically non-random (Fig. 3).

In relatively early embeddings, where broad genus and sex differences predominate, females of birdwing species from the two major genera *Ornithoptera* and *Troides* form a large central cluster with males around the periphery (Fig. 1) and mean, median and maximum inter-species distances are all higher for males than females (*n* = 595, Kruskal–Wallis, two-tailed *p* < 0.001, results consistent across 10 epoch embeddings from three independent ML training runs, Fig. S8). Similarly, in early embeddings, the average sexual disparity difference among birdwing butterfly species is positive, showing relatively high male disparity (e.g., epoch 10, mean = 0.13, 35 species).

However, analyses of disparity within 9 birdwing species groups^8,11^ (Fig. 2), learnt in joint embeddings by species label, reveal diversifications with contrasting trends in sexual disparity. This contrast is exemplified by the *O. paradisea* and *T. haliphron* species groups (Figs. 2–3), which show, respectively, extreme positive versus extreme negative values of the sexual disparity difference, showing respectively, higher male than female disparity, and vice versa. Multiple embeddings comparing the statistical repeatability of key results across training runs (which varied in run parameters such as the image batch sampling) and run stop points (explored effects of varying training time) showed that the signs of these sexual disparity differences were consistent across all of 15 sequential repeatability test embeddings, sampled from training epochs between 10 and 2990 from 3 independent ML training runs. The *O. priamus* and *O. paradisea* species groups exemplify an evolutionary pattern of high male disparity. For example, while females of the *O. paradisea* species group are closely clustered with each other, and with those of the *O. priamus* and *O. victoriae* groups, their males have diversified into comparatively distinct phenotypic sub-clusters, including one containing some of the most extreme morphologies among birdwing butterflies (Figs. 2 and 4). In contrast some (but not all) *Troides* species groups show an inverted pattern of visually similar males with relatively disparate females, exemplified by the extreme negative sexual disparity difference of the *T. haliphron* and *T. amphrysus* species groups.

On two grounds, we can reject null hypotheses of embedded phenotypic variation that is random with respect to evolutionary diversification among birdwing butterflies (i.e., the collective and distinct components of evolutionary history in males and females of the diversifying species), specifically null hypotheses of no sexual dimorphism and no inter-species correlation in embedded phenotype (diagram, Fig. 2c). First, phenotypic evolution that was random with respect to sex can be rejected overall, based on the sexual structure of phenotypic similarity (e.g., *p* < 0.001, above, Fig. S8) and non-zero sexual disparity differences observed within species groups. Second, random differences in sexual disparity among species samples can be rejected due to the non-random overlap between embedded phenotypic distance and independent genetic distance (*p* < 0.001, above; Fig. S6).

### Male and female contributions to inter-species disparity

Variation between males of birdwing butterfly species is generally more salient and distinctive to the ML network than that between females (Figs. 1, 2 and 4). Particularly, in relatively early embeddings the sexual disparity difference is consistently and very significantly positively correlated with inter-species phenotypic distance between males, but not consistently correlated with distance between females (Fig. 4a, male Spearman correlation *p* < 0.001, *r* ≥ 0.38 across 3 independent training runs, Fig. S9). In other words, as we consider more visually dissimilar species pairs there is a greater tendency for the disparity to be greater among their males than among their females. This pattern is consistent with the observed early embedding structure dominated, overall, by high male disparity (Fig. 1b) and male phenotypic distinctiveness (Fig. 4a–e).

### The evolution of sexual disparity

In genus *Troides*, including in species groups where female images were observed to be more phenotypically disparate than males such as the *T. haliphron* group, dimorphic male characteristics, including bright and contrasting wing colour patterns, are represented in the studied photographs (Figs. 2 and 4), in addition to, and not replaced by, other signalling modalities or aspects of phenotype (SI), as has been suggested in male antbirds, for example^27^. However, embedded male phenotypes are comparatively close in genus *Troides* (e.g., Fig. 2). Alongside learnt variation in wing phenotypes among females (which are primarily brown, with, for example, variable white and yellow patches and spots, Figs. 2, 4), there is higher female than male disparity in some *Troides* species groups (Figs. 2–3). This is contrary to some previous suggestions, for example based on rates of colour evolution inferred from illustrations of European butterflies^48^, that there is ‘no indication that it is common in butterflies for dichromatism to evolve due to female-limited chromatic evolution under natural selection, as argued by Wallace’^48^. Indeed, machine learning on comprehensive samples of whole-organism photographs adds to the previously rare examples of higher female than male variation in visible phenotypic traits beyond size, such as colour, shape or number of features^6,27,31^, showing that female variability learnt from visible phenotype can dominate observed phenotypic diversification in species groups with comparatively static male phenotype, as predicted by Wallace to result from relatively strong, divergent natural selection on females^5^. In contrast, in other species groups, particularly in genus *Ornithoptera*, we observe inverted evolutionary patterns, with high machine-learnt male disparity and extreme male phenotypes, predicted by Darwinian sexual selection^1,49^.

### Testing predictions of selection

The ML embedding method we use here is not a causal machine learning method (one that formally incorporates a causal model of the studied system^50^). Consequently, additional phylogenetic comparative analyses^51^, which test phylogenetic distributions of continuous biological trait data against models of evolutionary selection, were applied to the machine-learnt measures of phenotypic similarity and an analytically distinct, time-calibrated reference phylogeny of overlapping birdwing species^9^. For ML visual similarity learnt from separately labelled male and female photographs, a multi-peak Ornstein-Uhlenbeck (OU) model received strong Akaike information criterion (AIC) support over null hypotheses of evolution by pure Brownian motion or with a single OU peak for both males and females, across 10 repeated embedding samples (Fig. S10). Convergent evolution in sympatry is a prediction of shared natural selection^51^ and a corollary of Wallace’s prediction of divergent female defensive colour patterning in different habitats (to the extent that shared biogeography reflects shared habitat). Convergence within an ML embedding, comparable to ‘ecomorph’ convergence among general multivariate biological trait data^51^, was not detected between females from genera *Troides* and *Ornithoptera* where biogeographic ranges of birdwing genera overlap among species from New Guinea^8,12^, as opposed to allopatric species (specifically in *T. amphrysus* and *T. oblongomaculatus*, as opposed to other *Troides* species allopatric with New Guinean *Ornithoptera*, Fig. S10). Rather, phylogenetic comparative analyses identified a single selective regime for females of *Troides* within and outside their range overlap with *Ornithoptera*, and inferred that the disparity of females within and between *Troides* species groups (observed to be high relative to that of corresponding males) crosses one best-fitting selective regime (Fig. S10).

Exaggeration^49^, and potentially ^52,53^ diversification, of secondary sexual traits is a key prediction of sexual selection, as opposed to other possible factors, particularly in sexually dimorphic traits^25^ and sympatric species. Selective model testing^51^ returned a significantly higher number of OU selective regimes for males than females (Wilcoxon *W* = 55, *p* = 0.002, separate sex ML labels, number of best-fitting convergent regimes not significantly higher for females than males, Wilcoxon *W* = 12, *p* = 1, Fig. S10). Tests for variation between genera *Ornithoptera* and *Troides* in the rate of evolution across all image embedding axes (with separate sex labels) showed that rates were, on average higher in *Troides* for females (proportionate difference mean = 0.8), versus *Ornithoptera* for males (mean = 1.3, proportionate rate difference, *Ornithopetera*/*Troides*, 10 repeated embedding samples, paired *t* test *t* = −3.232, *p* = 0.018; Fig. S10). Therefore, machine-learnt phenotypic diversity, within and between birdwing genera, is correlated with variation in the number, breadth, and decoupling of inferred selective optima for the two sexes. These differences are associated with female investment in a smaller number of larger eggs in *Ornithoptera*, compared with *Troides*^54^, increasing asymmetries between male and female reproduction^2,21,55^.

### Biogeography and sexual diversity

Different models of sexual selection disagree on the likelihood of achieving stable coexistence of multiple sexually selected traits^49,52,53^. Consequently, two empirical questions remain open: the importance, in the real world, of allopatry in the diversification of sexually dimorphic phenotypes, and the extent of sexual variation in sympatric speciation^53,56,57^. The machine-learnt visual diversity among ornithopteran males includes phenotypically distinctive species, such as *O. croesus*, *O. aesacus* and *O. priamus*, for which allopatric island isolation accompanies the diversification of sexually dimorphic male traits (Figs. 2, 3, 4e, f). However, also present are species, notably the New Guinean *O. paradisea* group, exhibiting sexual dimorphism, high male phenotypic diversity, and sympatric geographic ranges (Figs. 2, 3, 4g).

### Darwin versus Wallace: sexual or natural selection

Wallace specifically argued for contributions to inter-species diversity from either males or females, examples of species with highly variable, ‘protective’ colouration in females but not males and a ‘tendency in the male of most animals—but especially of birds and insects—to develop more and more intensity of colour’, all evolutionary patterns recovered by this analysis^5^. However, Wallace also countered Darwin’s^1^ hypothesis of sexual selection for the evolution of sexual dimorphism in visual signalling traits, based specifically on scepticism of female mate choice^5,58^. Instead, Wallace primarily advocated natural selection mechanisms in promoting sexually dimorphic variation with a secondary role for a form of indirect sexual selection without ornamental trait preferences among females^5^. Sexual selection remains a highly active and debated research area^58^. However, the years of research since the origins of evolutionary theory have increased the evidence for female (and male^59^) mate choice^58^, including in insects and specifically among birdwing butterflies (in which, for example, female rejection of male courtship has been observed^12^, alongside elaborate male display behaviours, SI Supplementary Note 1). The further development of sexual selection theory, including runaway models^49^, also provides specific theoretical mechanisms for both the tendency towards male phenotypic exaggeration hypothesised by both Darwin^1^ and Wallace^5^ and its diversification, which Wallace could not explain, except by possible effects of individual variability ^5^. In light of our analysis, we can also consider how the patterns of high female disparity we recover relate to Wallace’s hypotheses on natural selection^5^. In discussing sexually dimorphic protective colouration among females, for example, Wallace hypothesised that ‘natural selection is constantly at work, preventing the female from acquiring [the] same tints [as the male], or modifying her colours in various directions to secure protection by assimilating her to her surroundings’ and (with regard to birds) ‘the different amounts of colour acquired by the females have no doubt depended on peculiarities of habits and of environment, and on the powers of defence or of concealment possessed by the species’. These statements link variability among female phenotypes to adaptation to different habitats, consequently predicting greater similarity of phenotype in the same habitat, via stasis or evolutionary convergence. However, evolutionary model-testing on our machine-learnt phenotypic trait values was unable to detect, among embedded female images, overall phenotypic convergences in the same geographic region alongside divergence of selective regimes in different biogeographic regions. Instead, recovered female and male variation was characterised by varying extents of divergence, with a greater diversification of selective regimes inferred among males versus broad selective regimes associated with genera of females (Fig. S10). This provides evidence for contrasting selection in males and females, potentially resulting from diversification of female preferences and male phenotypes, under sexual selection, particularly in *Ornithoptera*, versus more subtle diversification of female protective colour patterning (potentially combining crypsis and aposematism, SI) within broader inter-species natural selection regimes.

Overall, therefore, we demonstrate that machine learning can recover rich and complex patterns of phenotypic variation. These include both salient male variation, predicted by Darwin^1^ under the female mate choice that contemporaries doubted^5^ and, under-studied^31^, female variation, with this case supporting some, but not all, of Wallace’s predictions under natural selection^5^. We have noted that these contrasting patterns in the evolution of disparity are correlated with observed differences in reproductive input between birdwing genera^54^. Our results, therefore, suggest that differences in the dynamics of natural versus sexual selection within a broader evolutionary radiation can result in sexual dimorphism, inter-species phenotypic variation, and a diversity of underpinning evolutionary patterns. Looking forward, machine learning offers opportunities to measure phenomic diversity, and test hypotheses on its evolutionary causes, at unprecedented scales.

## Methods

### Recuration

Birdwing butterflies (Lepidoptera: Papilionidae: genera *Ornithoptera*, *Trogonoptera* and *Troides*) were chosen for exhaustive specimen imaging by the Natural History Museum, London (NHMUK) due to the extensive collection, brightly coloured specimens, relatively well-established taxonomy^10,11^ and large specimen size which facilitates study. In preparation for digitisation, a comprehensive recuration of the birdwing specimens at the Natural History Museum (NHMUK) was carried out, collating specimens from across the several collections and arranging them taxonomically into a consolidated collection, arranged into the three genera, 35 species OTUs (operational taxonomic units) and 131 subspecies (online on the NHMUK Data Portal). Specimen RGB photographs were then taken by the Digital Collections Programme digitisers, covering the entire (NHMUK) birdwing collection. Original specimen photographs and accompanying metadata are available through the NHMUK Data Portal at https://data.nhm.ac.uk/dataset/collection-specimens.

### Photographic dataset

Photographic data were screened for this study to include only specimens that were adults, possessed all four wings, and were photographed in both dorsal and ventral view. Original photographs, which include additional items such as specimen labels, were cropped^44^ to include only the butterfly specimen padded with a 1-pixel border, using image segmentation in MatLab. Images were rescaled (with fixed aspect ratio) to a uniform pixel resolution of 64 pixels high for ML analyses^35^, in order to standardise and reduce the problem size, corresponding memory and training time requirements, and potential for model overfitting in image comparisons via the CNN. Operational taxonomic units at the species level (e.g., including resolution of suggested species synonyms and treatment of potential subspecies for the purposes of analysis) were based on the NHMUK taxonomy for each image listed on the NHMUK Data Portal. The image dataset used in our machine learning analyses is provided (Supplementary Data 3), with corresponding taxonomic label data (Supplementary Data 1). Total numbers of images of female and male birdwings were respectively 7840 and 8894. Image sample sizes were respectively, for the 35 OTUs: *T. aeacus* 1044, *O. aesacus* 24, *O. alexandrae* 78, *O. allottei* 6, *T. amphrysus* 1060, *T. andromache* 110, *T. brookiana* 894, *O. chimaera* 168, *T. criton* 208*, T. critonides* 140, *O. croesus* 374, *T. cuneifera* 266, *T. darsius* 436, *T. dohertyi* 136, *O. goliath* 200, *T. haliphron* 874, *T. helena* 2608, *T. hypolitus* 516, *T. magellanus* 60, *O. meridionalis* 32, *T. minos* 418, *T. miranda* 234, *T. oblongomaculatus* 978, *O. paradisea* 238, *T. plato* 56, *T. prattorum* 38, *O. priamus* 4136, *T. rhadamanthus* 88, *T. rhadamantus* 296, *T. riedeli* 46, *O. rothschildi* 46, *O. Tithonus* 92, *T. trojana* 64, *T. vandepolli* 178, *O. victoriae* 592.

### OTU evaluation

Operational taxonomic units were included for analysis based on the specific epithet for each given image included in this dataset listed on the NHMUK Data Portal. OTUs that subsequently showed lowest phenotypic distinctiveness due to a near neighbour in the embedding (see methods below) include *T. critonides*, in line with its previously suggested synonymy with *T. criton criton*^60^. This indicates that ML embedding distance can be used to quantitatively assist further taxonomic evaluation of sampled specimens or populations, here from historical museum collections.

### Genetic dataset

The extent to which inter-species distances within machine-learnt embeddings of birdwing butterfly photographs (e.g., learnt using different algorithms or parameter values) contain structure that is meaningful with regard to biological evolution was tested by comparison against calculated inter-species genetic distances. Published gene sequences for birdwing butterflies were downloaded from the GenBank repository, based on collated accessioning information^8^ (Supplementary Data 2). Sequences were available for four genes with minimum coverage of 33 specimens: COI (mitochondrial cytochrome c oxidase subunit 1), 120 specimens; 16 S (mitochondrial 16 S ribosomal RNA), 59 specimens; ND5 (mitochondrial NADH dehydrogenase subunit 5), 33 specimens; EF-1α (nuclear elongation factor 1-alpha), 68 specimens. Collectively, these covered 177 butterfly specimens and 30 of the 35 birdwing OTUs used for our photographic dataset.

### Genetic analyses

DNA sequences were aligned within each gene using the programme MUSCLE^61^. The best-fitting model of DNA substitution was tested for each gene using the programme jModelTest 2.1.7^62,63^ with a fixed BIONJ tree, 3 substitution schemes for likelihood calculations, and the AIC selection criterion. Selected substitution models used for further analysis were: COI, GTR + G (likelihood -lnL = 12831, AIC = 26154), 16 S GTR + I + G (-lnL = 1971.1098, AIC = 4192); ND5, HKY + I + G (-lnL = 4203.2407, AIC = 8544); EF-1α, K80 + I + G, equivalent to HKY with equal base frequencies (-lnL = 2412, 5096).

Bayesian phylogenies of sampled gene sequences were first reconstructed from each gene partition using BEAST, with both a strict molecular clock and relaxed lognormal clock, which allows substitution rate variation between branches (BEAST input xml file, Supplementary Software 2). A lognormal mean substitution rate prior was provided of 0.01909 substitutions per site per million years, based on the published average substitution rate estimated across 10 genes, including the 4 used in this study, on a fossil calibrated phylogeny of 18 genera of Papilioninae^64^. Each clock analysis (strict/relaxed) was run using two independent MCMC chains of length 10,000. The effective sample size (ESS) of each analysis was examined, and model comparison was performed using Tracer based on combined logs from the two chains, with a 10% burn-in (a standard practice, which discards trees from the earliest part of the tree search which may not yet have achieved representative likelihoods). Comparison of model likelihoods was conducted, supporting a relaxed molecular clock over a strict clock (likelihood relaxed = −21633, strict = −21779), therefore the genetic trees from the relaxed clock runs (with posterior ESS = 146) were used in further analyses.

For comparison against inter-species distances within our phenotypic spatial embeddings, we reconstructed species-level genetic phylogenies using the Bayesian multi-species coalescent method with the programme Astral^65^. Astral reconstructs the most probable species phylogeny given an input set of trees from different parts of the genome, which, due to standard phylogenetic processes, such as gene coalescence through lineages, rapid divergence, polytomous speciation, or hybridisation can show differing phylogenetic histories^66^. In order to provide a measurement context for comparisons between phenotypic and genetic phylogenetic signals, as described in further detail below, we measured the distribution of similarity among likely genetic trees by reconstructing a set of 1000 species trees, each reconstructed by sampling one tree from each of the 4 included genes from the Bayesian MCMC chains (post-burn-in).

### Machine learning embedding method

We made improvements to the architecture of the original ButterflyNet^35^ CNN (ButterflyNet 1.2, Supplementary Software 1). We removed the classifier part of the original architecture^35^ so that embedding structure is based solely on triplet-training loss, facilitating exploration of the effects of triplet-loss training duration on the correlation between inter-OTU Euclidean distances in the image embedding (a proxy for whole-specimen visual similarity) and independent genetic distances based on housekeeping genes. We use Euclidean embedding distance measures since, similar to other triplet implementations^44^, our architecture is based on an Euclidean triplet-training loss function^35^. This loss function was additionally modified to a margin loss (with a range of margin values). We quantitatively evaluated the relationship between network training time and the correlation between average OTU embedding distances and genetic distances. We also performed data augmentation using random-affine transformations of the original data using the RandomAffine function from the PyTorch^67^ torchvision package (incorporating random image translations, scaling, and shears). Data were presented to training in randomised order, relative to the initial ordering of the joined data (using a numpy random seed of 12345, Supplementary Software 1). A sampling step was included in order to mitigate the potential effects of imbalance in the numbers of images of each species in the photographic dataset on the distribution of inter-species distances in the embeddings. During the sampling of image triplets for the triplet-training ML method (which presents two images of the same species and one image of a different species to the CNN), we first sampled the species for the two images that were to be the same (sampling, with equal probability, one species from the OTU list), then sampled two images from the selected species (sampling images, with equal probability, from those available for the given species). This ensured that each training batch sampled the species to be the same with uniform probability from the list of included species (an additional uniform sampling option for the species selected to be different in each triplet is also available in Supplementary Software 1). Data required to replicated machine learning analyses and a representative trained ML model are provided as Supplementary Data files 3–6.

### Calculation of average embedding distances

Biological subsets of the photographic dataset, including image surface (dorsal/ventral), biological sex, and species (based on NHMUK records), were used to calculate average embedding locations across all photographs within each subset, across the 16,734 images in the dataset, and to calculate the corresponding Euclidean distances between subset pairs.

### Pixel correlation analyses

For comparison with the ML analyses, a supplementary analysis was conducted based on simple comparisons of the Pearson correlation coefficient between values of overlying RGB pixels between identically scaled, sampled image pairs (Supplementary Software 3). Compared pixels were therefore those with the same x, y coordinates in sampled, uniformly scaled images, compared between corresponding RGB colour channels. Comparisons were conducted at three levels of image resolution: 64, 32, and 16 pixels high. 1000 images were randomly sampled for pairwise comparisons from the full image dataset of 16,734 images. Prior to pixel comparison, ventral images were flipped horizontally, to match the orientation of corresponding dorsal views. In each pairwise image comparison, the second sampled image was rescaled to match the dimensions of the first sampled image. For comparison with results based on our ML method, phylogenetic trees were reconstructed based on average pixel correlation values between images sampled from species. To prepare an input suitable for phylogenetic reconstruction (see methods below), a pairwise inter-species distance matrix was constructed based on the mean pixel correlations for compared images sampled from the appropriate species. The matrix was then scaled by subtraction from one, such that a pixel correlation of one would correspond to a distance of zero for phylogenetic reconstruction. All phylogenetic trees were pruned to the same taxon set as the genetic trees and given the same rooting (to *T. brookiana*) prior to tree comparisons. Since phylogenetic tree similarity measures such as Euclidean tree distance are sensitive to branch lengths, an additional analysis was conducted for comparison, in which input distance matrices (based on either pixel correlations or embedding distance) were individually rescaled to a proportion of their maximum prior to phylogenetic reconstruction and comparison.

### Phylogenetic reconstruction

Reconstruction of phylogenies based on ML distances between images is a new method of phylogenetic reconstruction^35,36^, which presents new opportunities and questions. For example, we specifically address here the extent and type of phylogenetic information captured, relative to analytically independent phylogenetic signals from genomic samples for shared taxa^35^, given ML training time. In traditional morphological character analysis a human observer generally views an entity (e.g., a biological individual) and attempts to break up an observed phenotype into encoded characters^68^. The main task of phylogenetic algorithms is usually to reconstruct one or more phylogenetic trees from these characters e.g., reconciling conflict between them. In comparison, the ML triplet embedding is trained to give each individual image a location in a multidimensional space that collectively represents the information learnt from visible phenotype and in which, inter-image distances are defined to be Euclidean^35,43,44^. The main task remaining for phylogenetic reconstruction is then to summarise the hierarchical relationships between images, or groups of images, in the overall embedding, for which we used the neighbour-joining algorithm^35,69^. The mean embedding location of the photographs of each species OTU was first used to calculate a square-form pairwise inter-species Euclidean distance matrix, using the Python Scipy package^70^. A neighbour-joining phylogeny was then reconstructed from the phenotypic distance matrix (using the Python biotite.sequence.phylo sub-package^71^, Supplementary Software 1). Neighbour-joining phylogenies were reconstructed based on average embedding distances between species, considering all images and, for comparison, considering female images or male images only. Clade depths were calculated from the highest terminal in a clade to the most recent common ancestor of the clade using the software package Mesquite 3.11^72^.

Phylogenetic signals reconstructed from the machine-learnt embeddings were quantitatively compared to phylogenetic signals from independent genetic data (where phylogenetic signal is defined, generally, as the information on relative relatedness between OTUs from a given data partition^47^). During ML training, the Pearson correlation coefficient between pairwise matrices of inter-species embedding distance and average genetic distance (which can be rapidly calculated) was reported every 10 training epochs (Supplementary Software 1). This was supplemented by subsequent analyses based on phylogenetic reconstruction from output embedding distances. Since we were interested in further use of evolutionary distance measures based on the phenotypic embedding, phylogenetic trees were compared using a statistic that considers both tree topology and branch lengths, the Euclidean distance (branch-length distance^73^) tree similarity measure, calculated using the Python Dendropy package^74^. All trees were given a common rooting (*Trogonoptera brookiana*) prior to the calculation of tree similarity statistics.

To evaluate the extent to which machine-learnt measures of visual similarity are correlated with independent genetic signals of evolution we assessed statistical distributions of pairwise incongruence. Where genetic data are an appropriate benchmark, measures of incongruence can considered a measure of ML tree quality, like, for example, the incongruence length difference test used previously to evaluate trees based on different genetic loci^75,76^. A tree representing a given genetic locus itself provides a specific phylogenetic signal (its implied levels of relatedness among the specific OTUs^47^), which may deviate from other signals, including those from ML, for a range of reasons, including inter-gene conflict resulting from normal genetic processes^66^, or partial information. Given this, we measured pairwise incongruence between a tree based on a given embedding and a sample of species tree based on published genetic data, relative to the pairwise incongruence among the genetic data themselves. This assesses the extent of congruence between ML and genetic signals relative to the extent of congruence present among the evolutionary histories of available genetic loci. Where such ML-gene incongruence is low, this demonstrates agreement between the machine-learnt measure of visual similarity and genetic evolution. Here, we compare ML phylogeny against published housekeeping genes, which have been previously used to reconstruct the history of speciation and biogeographic radiation among birdwing butterflies^8^.

### Phenotypic distinctiveness

Measures of phenotypic distinctiveness were calculated from the pairwise embedding distances, using the pairwise, minimum, and mean distance in the ML image embedding of each species from all others. The minimum distance criterion ranks phenotypic distinctiveness such that the most distinctive species are those that have the greatest centroid distance from any other species. The mean distance criterion ranks phenotypic distinctiveness such that the most distinctive species are those that have the greatest average centroid distance from all other species.

### Comparative analyses

Comparisons of statistics calculated from embedding distances were conducted across the whole dataset, among genera, and within 9 species groups. These are groups of closely related species, previously proposed based on qualitative assessments of morphology^11^, taxonomy, and genetic phylogeny^8^. Phylogenetic comparative tests of predictions from different selective models for the phylogenetic distribution of machine-learnt phenotypes were conducted using the R package SURFACE^51^ against a distinct, published reference phylogeny (IQ tree of ref. ^9^) for 30 overlapping OTUs. Embedding locations for the phylogenetic comparative analyses, conducted separately for each sex, were the mean OTU locations on each of 64 embedding axes, for embeddings with separate male and female species labels (allowing male and female images of the same species to be located separately in the embeddings). Given these trait values and the reference phylogeny, SURFACE allows tests of alternative evolutionary selective models (comparing model likelihoods: higher values indicate higher support) given their number of parameters, via their AIC values (Akaike Information Criterion, lower values indicate higher support)^51^. A null hypothesis of pure Brownian motion was tested against single-peak and multi-peak Ornstein-Uhlenbeck models, which model the presence of adaptive peaks. The resultant best-fit model and AIC support were recorded in addition to the number of best-fitting selective regimes, and the number of these inferred to be convergent on the phylogeny. A priori hypothesis tests for variation in the rate of evolution across the 64 image embedding axes on a distinct reference phylogeny were conducted for genera *Ornithoptera* versus *Troides* using the R package motmot^77^. Separate hypothesis tests were conducted for females and males using image locations in embeddings trained with separate male and female labels.

### Calculation of sexual disparity differences

Biological groups, such as birdwing butterflies, where reproductive investment, e.g., gamete size, is greater in females, courtship displays are shown by males, and mate choice including potential mate rejection is observed in females (see SI Supplementary Notes 1–2) provide test cases for the maximal effects on phenotypic diversity (disparity) of sexual selection on male phenotype from female mate choice^1^ versus relatively stronger natural selection on the phenotypes of females^5^. The relative extents of male and female image variation were summarised using a statistic, which we call the sexual disparity difference. To calculate this, we first separately calculated the average distances between subsets of males and subsets of females (we calculated subsets within each sex under two conditions, all images and dorsal/ventral images). Average Euclidean distances were then calculated between all subset pairs. This produces one inter-species pairwise distance matrix for each sex. We then calculated the sexual disparity difference as the difference between the male and female pairwise distance matrices. This results in a matrix of sexual disparity difference measures, one measure for each possible pair of subsets (e.g., species 1, dorsal surface images versus species 2, dorsal surface images). If the sexual disparity difference is positive, for a given data subset, it means that the distance between male subset centroids is greater than the distance between female centroids for the corresponding subsets. If the sexual disparity difference is negative, it means that the distance between female subset centroids is greater than the distance between male subset centroids. If the difference is zero, it would mean that centroid distances between males and females of the corresponding subsets were equal.

### Statistics and reproducibility

Statistical analyses were conducted using the Python Scipy Stats package^70^ and PAST^78^. Normality tests used the D’Agostino and Pearson omnibus normality test^79^ or Shapiro-Wilk test. Where non-normal distributions were observed, non-parametric statistical tests were used for further analyses. All tailed tests were two-tailed. The birdwing butterfly image dataset analysed in this study comprised 16,734 photographs.

### Reporting summary

Further information on research design is available in the Nature Portfolio Reporting Summary linked to this article.

### Supplementary information

Supplementary Information

Description of Additional Supplementary Files

Supplementary Data 1–2 and 7–10

Supplementary Data 3

Supplementary Data 4

Supplementary Data 5

Supplementary Data 6

Supplementary Software 1

Supplementary Software 2

Supplementary Software 3

Reporting Summary

## Data Availability

All photographic data used in this study and metadata are publicly available at https://data.nhm.ac.uk/dataset/collection-specimens. Supplementary Data provided with this study are 1 image label data and 2 accession numbers of publicly available genetic data used in this study. 3 processed photographic data used in machine learning and additional analyses, 4 taxonomic labels formatted for machine learning analyses, 5 representative trained machine learning models, 6 matrix of genetic distances for comparisons in machine learning, 7-10 source data for Figs. 1–4, respectively. Supplementary information, data, and software are publicly available at the Dryad data repository^80^.

## Code availability

Computer code used to perform machine learning analyses, and trained ML models, are provided as Supplementary Software 1 (ButterflyNet Version 1.2). Aligned genetic data, and accompanying analytical parameters, are provided as a BEAST xml input file in Supplementary Software 2. Computer code for pixel correlation analyses is provided as Supplementary Software 3. Supplementary Software is available via Dryad^80^.
