# Supplementary material for: Male and female contributions to diversity among birdwing butterfly images
Source: Commun Biol. 2024 Jul 1;7:774. doi: 10.1038/s42003-024-06376-2 (PMC11217504; doi:10.1038/s42003-024-06376-2)
Supplement: Supplementary file 1 — Supplementary Information [file 42003_2024_6376_MOESM1_ESM.pdf]

Male and female contributions to diversity among birdwing butterfly images

Jennifer F. Hoyal Cuthill[1,*], Nicholas Guttenberg[2] and Blanca Huertas[3]

[1]School of Life Sciences, University of Essex, Colchester, UK.

[2]Cross Labs, Cross Compass Ltd, Tokyo, Japan.

[3]Department of Science, Natural History Museum, London, UK.

[*]Corresponding author E-mail: j.hoyal-cuthill@essex.ac.uk

Supplementary Information

Biology, natural history and behaviour of birdwing butterflies - Supplementary Note 1

While the protected status of birdwing butterflies restrict the range of experiments which may be performed on the group[1], a number of detailed studies of morphology have been performed on small samples of birdwing species and specimens, many of these focusing primarily on the morphology only of males[1,2]. These studies have revealed a range of mechanisms producing the spectacular colouration among these butterflies, including pigments unique to papilionid butterflies, some of which have been shown to be fluorescent[3], as well as species or sub-specific structural scale morphologies that generate iridescence and combined effects from pigmentary and structural colouration[1].

Birdwings are primarily species of primary tropical forest where their foodplant vines grow, though some species extend to disturbed forest or parks if their larval foodplant is present[4,5]. Given their dependence on tropical forest, birdwings are at conservation risk from a range of environmental pressures including logging and framing as well as illegal trade prompted by their visual attractiveness.

Several authors have suggested the opinion that female birdwing butterflies are, in general, less brightly coloured than males[3]. However, it has also been noted that birdwing

butterfly larvae feed on toxic foodplants and both male and female adults can have some wing areas with brightly reflective colouration, indicating the potential for aposematic signalling to potential predators[1]. Birdwing butterfly larvae, feed on hostplants of the family Aristolochiaceae (birthworts) which produce highly toxic aristolochic acids (e.g. *O. primus, Aristolchia*)[4–6]. However, larvae[6] and adults[7] have been observed to be attacked, and sometimes eaten, by predators including birds as well as ants and wasps, while other local potential predators include spiders, frogs[8] and lizards[6]. Furthermore, aspects of both male and female dimorphic colouration, visible on the wing, have been noted to be recognisable to a human observer (e.g. in *Trogonoptera brookiana*[8,9]). In general, it is also hypothesised that aposematic colour patterns may have additional cryptic or camouflage functions, for example when viewed from a greater distance. Male birdwings have also been observed in territorial flights (e.g. *Trogonoptera brookiana*[9], *Troides dohertyi*[5]) sometimes involving direct physical contact, indicating the potential importance of visual recognition of male conspecifics.

Differences in behaviour between male and female birdwing butterflies have been observed, with relevance for the evolutionary selection pressures on sexual dimorphism. Birdwing butterflies exhibit elaborate courtship displays, although mating with newly emerged females with more limited male courtship has also been observed (e.g. *Ornithoptera priamus*[5] and *Troides oblongomaculatus*[10]). Both males and females can participate in courtship flights[8,9]. While comprehensive biological records for birdwing butterfly species are lacking[8], there are isolated natural history records for both genera *Trogonoptera* and *Troides* of courtship displays in which the male maintains a flight position above a flying female, the female therefore viewing the male dorsal surface, and vice versa, e.g. in *Trogonoptera brookiana*[8,9] and *Troides darsius*[8]. Footage of male courtship display in *Ornithoptera priamus*, for example, shows that the males move into and out of a position and wing inclination in which the dorsal surface of the hindwings is in front of the head of the

female. Male *Troides* (e.g. *T. oblongomaculatus*) possess scent (androconial) pouches which are engaged with female antennae via a flight manoeuvre in which the male flies against the female from below and behind[10]. Male *Ornithoptera* also possess similar hairs on the inner margin of the hindwing[10]. Structural analysis of the dorsal hindwing of the birdwing butterfly *Troides magellanus,* shows that the dorsal hindwing (which is pigmented in bright yellow, while the dorsal forewing is predominantly black) shows a flash of iridescence when viewed from at an angle close to the wing plane[3], producing structural colour variation which is visible in flight[8]. The authors of that study state that the female of this species, while possessing areas of bright yellow pigmentary wing colouration, does not exhibit iridescence[3]. A similar iridescence phenomenon is also visible in photographs of male *Troides prattorum*, and also noted to occur, to a reduced degree, in the female[8]. Video footage of birdwing behaviour also show examples in which a male of *Ornithoptera* flies in front of, and slightly above, a female perched below, with her wings held horizontally[11], or with her wings rapidly vibrating[12]. Footage of *Ornithoptera* courtship flights additionally shows the male ascending then dropping vertically to, or below, the female, alternately moving the ventral and dorsal wing surfaces above and below the viewing partner e.g. *Ornithoptera richmondia*[13]. Female birdwings have the observed capacity to refuse male courtship attempts, for example by dropping from flight to the ground and by extending their wings horizontally to prevent mating[5].

Unlike some other butterflies, birdwings have been observed to rest frequently with wings held horizontally, and during feeding, for example, wings can be held in part-closed v-shape[8], although Tutt notes that a female *T. brookiana* observed in temporary captivity was also seen to settle to rest with the wings closed, exposing only the ventral surface to view[9]. It is noticeable in photographs and video of living birdwings that the dorsal hindwings can be

comparatively visible in both *Troides* and *Ornithoptera*, for example undergoing less motion and/or being held at a wider angle than the forewings (e.g. field photos in [8]).

Natural versus sexual selection - Supplementary Note 2

There are multiple definitions of sexual selection and its distinctions from natural selection have been debated. However, one useful broad distinction is to group under sexual selection, variation in mating success resulting from interactions including mate choice and intra-sexual competition[14,15]. Natural selection then encompasses the remainder of selective pressures affecting fitness. Both natural selection and sexual selection can act on either sex[14–17]. It has been noted[18] that male variability has sometimes been automatically attributed to sexual selection. However, any variation in natural selection on males (e.g. resulting from sexually variable aspects of behaviour or ecology) can also be predicted to cause, or contribute to, evolution of male phenotypes. All else being equal, equal selection pressures predict equal phenotypes in both sexes[19]. However, where natural selection pressures acting on two sexes differ[20,21,18], their phenotypes may evolve accordingly, in either sex, or in both sexes. As known to Darwin[14], sexual selection can also act on either sex, with extent and outcomes affected by factors including relative energetic contributions to offspring production (in butterflies likely higher in females[18,20,22]), relationship between mating success and number of offspring[23,24], and the variation in extent of visual mate choice[25]. Overall phenotype is then expected to depend on the combined outcome of natural and sexual selection, their interactions[25] and constraints[26]. Key evidence supporting a role for sexual selection on male birdwing butterflies includes observations of female mate choice, including courtship rejection[5], alongside the sexually dimorphic, conspicuous and diverse wing colour pattern and shape phenotypes of birdwing males.

Image resolution	Statistics
Image resolution 64 pixels high<br>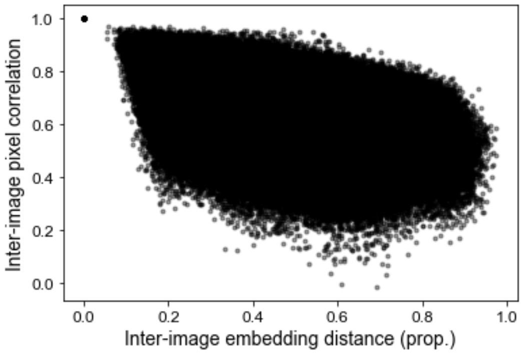	Omnibus normality test<br>n=1000000<br>Embedding distances (prop.)<br>statistic (s^2 + k^2) = 58429.30361806822<br>p < 0.001<br>Pixel correlations<br>statistic (s^2 + k^2) = 27614.027370443328<br>p < 0.001<br>Spearman correlation<br>corr.=-0.4472191002938924<br>p < 0.001
Image resolution 32 pixels high<br>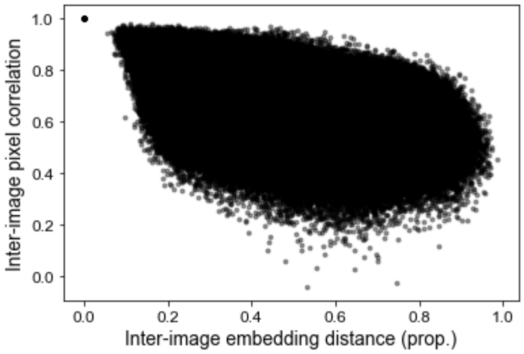	Omnibus normality test<br>n=1000000<br>Embedding distances (prop.)<br>statistic (s^2 + k^2) = 57864.60948609874<br>p < 0.001<br>Pixel correlations<br>statistic (s^2 + k^2) = 32366.108976292424<br>p < 0.001<br>Spearman correlation<br>corr.=-0.44773729955266983<br>p < 0.001
Image resolution 16 pixels high<br>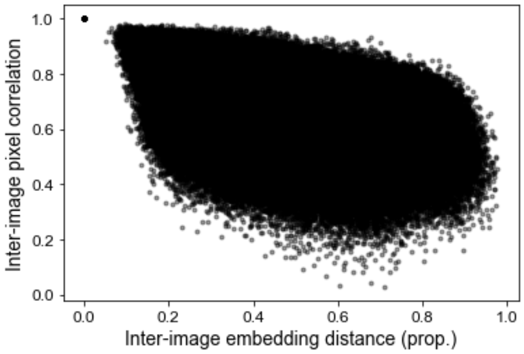	Omnibus normality test<br>n=1000000<br>Embedding distances (prop.)<br>statistic (s^2 + k^2) = 63099.818412084256<br>p < 0.001<br>Pixel correlations<br>statistic (s^2 + k^2) = 40464.5434844377<br>p < 0.001<br>Spearman correlation<br>corr.=-0.45385489431999987<br>p < 0.001

Fig. S1. Comparison between inter-image embedding distances and inter-image RGB pixel correlations. Each panel row shows plot and statistics for matched pairwise comparisons for 1,000,000 image pairs randomly selected from the full image dataset (of 16,734 butterfly photographs). There are significant negative correlations between proportionate inter-image distance in the embeddings (trained for 10 epochs) and the RGB pixel correlation coefficient for the corresponding image pair ($p < 0.001$, $n = 1{,}000{,}000$ randomly sampled image pairs). However, there is considerable scatter (correlation coefficient > -0.45), demonstrating that the information on visual similarity captured by the embedding is distinct from that captured by simple comparisons of overlying image pixels. Restricting pixel comparisons to image pairs from different species, and showing the same wing surface (removing, respectively intra-specific, and inter-surface variation) increases the correlation with embedding distances (correlation coefficient: 64 pixels -0.4681, 32 pixels -0.4445, 16 pixels -0.4658, 4 d. p., $n = 1{,}000{,}000$ image pairs).

Image resolution 64 pixels high<br>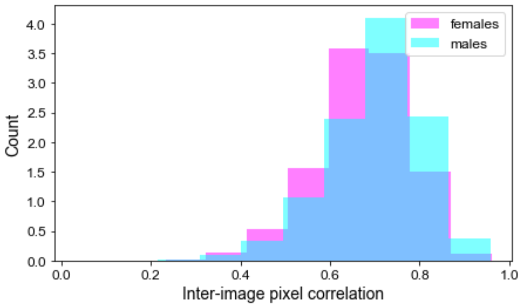 	Summary statistics<br>n = 227939<br>male mean = 0.7023686029525423<br>male median = 0.7157740653787693<br>male max. = 0.9580887069457072<br>female mean = 0.6727637509553436<br>female median = 0.6795767413403445<br>female max. = 0.9606063817447489<br>male-female mean = 0.0296048519971990<br>Omnibus normality test<br>female inter-species distances<br>statistic (s^2 + k^2) = 11383.01003598023<br>$p < 0.001$<br>male inter-species distances<br>statistic (s^2 + k^2) = 24583.130887945936<br>$p < 0.001$<br>Statistical test results<br>Kruskal Wallis test for equal medians (null)<br>H = 12017.45158799488<br>$p < 0.001$<br>Mann-Whitney test for equal distributions (null)<br>H = 25372501599.0<br>$p < 0.001$
Image resolution 32 pixels high<br>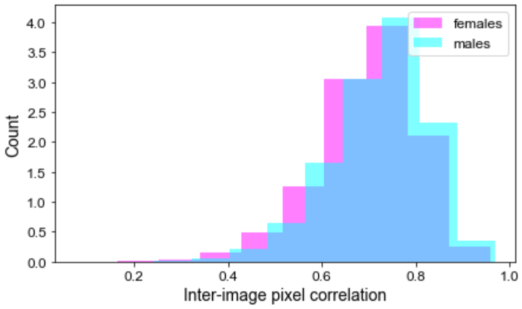 	Summary statistics<br>n = 232458<br>male mean = 0.7250180553835958<br>male median = 0.7379625165117691<br>male max. = 0.9688789828955299<br>female mean = 0.6976197697873574<br>female median = 0.7083434557563384<br>female max. = 0.9597155302107676<br>male-female mean = 0.02739828559623836<br>Omnibus normality test<br>female inter-species distances<br>statistic (s^2 + k^2) = 20284.86469168253<br>$p < 0.001$<br>male inter-species distances<br>statistic (s^2 + k^2) = 21398.449874991773<br>$p < 0.001$<br>Statistical test results<br>Kruskal Wallis test for equal medians (null)<br>H = 9211.226360011513<br>$p < 0.001$<br>Mann-Whitney test for equal distributions (null)<br>H = 26077724369.0<br>$p < 0.001$

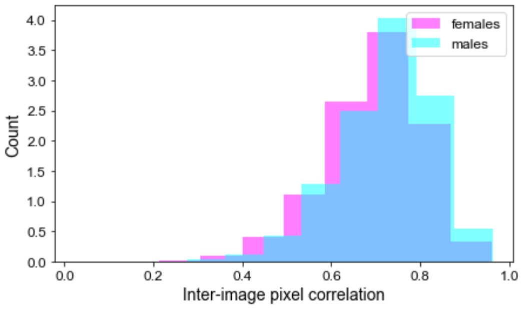	Summary statistics<br>n = 224658<br>male mean = 0.7253400299637963<br>male median = 0.7390193575311923<br>male max. = 0.9626496928953441<br>female mean = 0.6959166479870877<br>female median = 0.7069310893868774<br>female max. = 0.9619373716665244<br>male-female mean = 0.029423381976708574<br>Omnibus normality test<br>female inter-species distances<br>statistic (s^2 + k^2) = 15448.648535478493<br>p < 0.001<br>male inter-species distances<br>statistic (s^2 + k^2) = 20113.064826353693<br>p < 0.001<br>Statistical test results<br>Kruskal Wallis test for equal medians (null)<br>H = 9781.775827904394<br>p < 0.001<br>Mann-Whitney test for equal distributions (null)<br>H = 25944001036.0<br>p < 0.001

Fig. S2. Image pixel correlations for pairs of images that are both males versus both females. Each panel row shows plot and statistics for matched pairwise comparisons for randomly selected image pairs that meets the conditions that two images in a sampled pair must be of different species and the same wing surface (i.e. both images dorsal or both images ventral). These analyses show a small but significant increase in average pixel correlation for male versus female images, potentially resulting from differential effects of the sensitivity of pixel correlation to image feature translation (see Fig. S2). In contrast, in general, ML CNNS are insensitive to translation[27].

a) Pixel correlation (32 pixels): female images

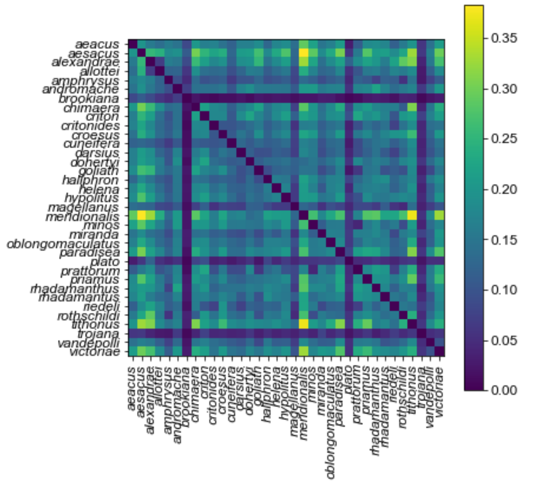

b) Pixel correlation (32 pixels): male images

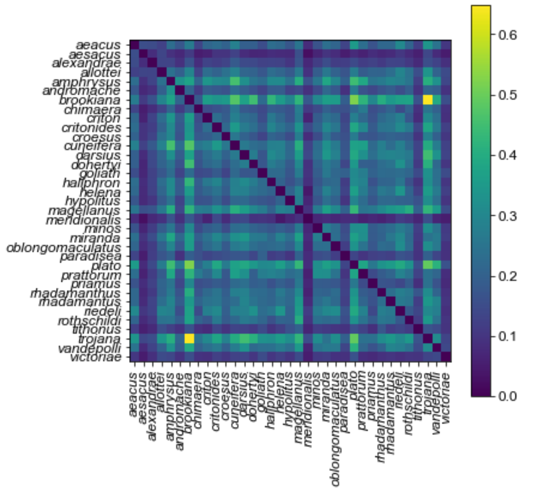

c) Pixel correlation complement (32 pixels): female

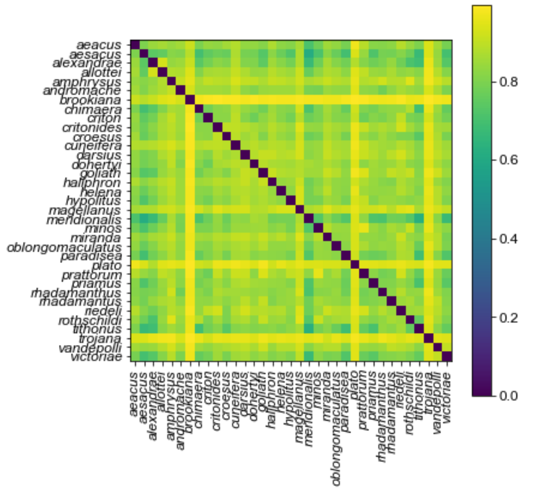

d) Pixel correlation complement (32 pixels): female

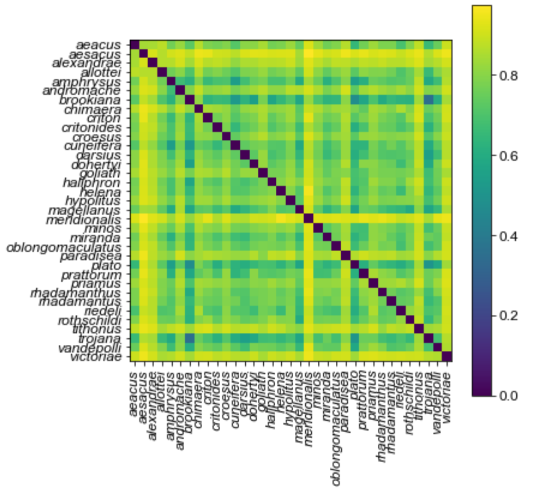

e) Embedding distance (10 epochs): female images

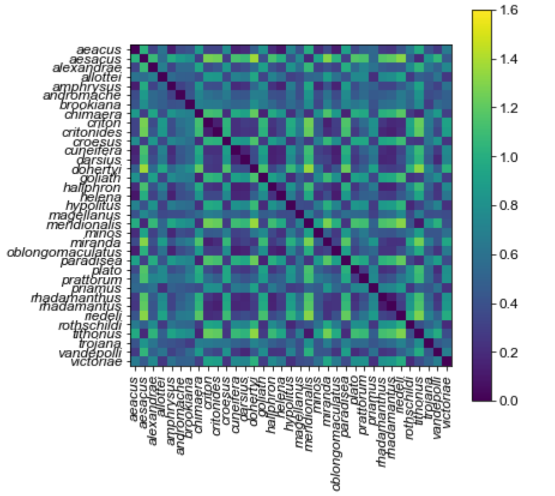

f) Embedding distance (10 epochs): male images

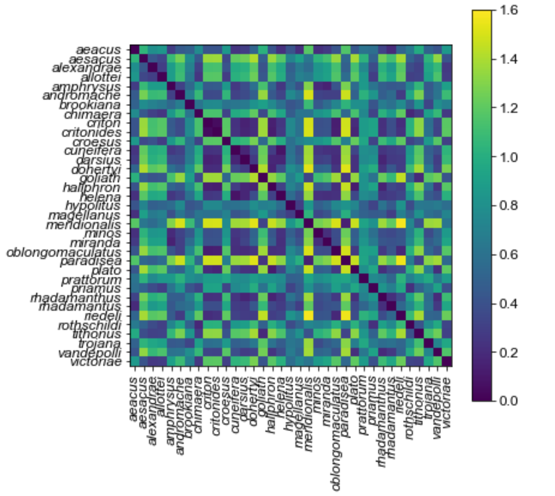

**g**) Pixel correlation (32 pixels): female images<br>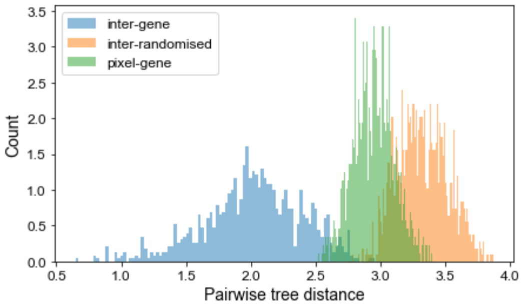 	minimum=<br>2.5228924623722335<br>maximum=<br>3.4039538757305077<br>mean=<br>2.94760708865942<br>median=<br>2.950171427319458<br>sample size=<br>9900
**h**) Pixel correlation (32 pixels): male images<br>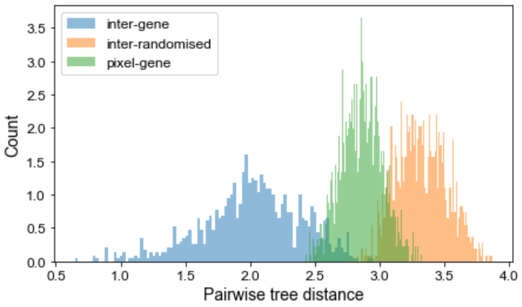 	minimum=<br>2.4302261370498535<br>maximum=<br>3.333513872855118<br>mean=<br>2.8605747520893905<br>median=<br>2.862877460110604<br>sample size=<br>9900
**i**) Embedding distance (10 epochs): female images<br>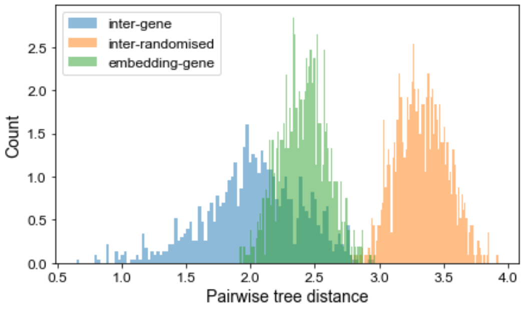 	minimum=<br>1.9155050921127632<br>maximum=<br>2.970263721675323<br>mean=<br>2.415181578829611<br>median=<br>2.4217065930074453<br>sample size=<br>9900
**j**) Embedding distance (10 epochs): male images<br>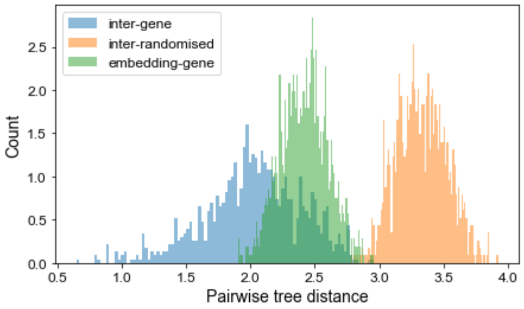 	minimum=<br>1.9065043240737847<br>maximum=<br>2.9623564987999766<br>mean=<br>2.417001562824732<br>median=<br>2.4223765474382564<br>sample size=<br>9900

Fig. S3. Comparison between image pixel correlation and ML embedding results for females versus males. a-b) Mean pixel correlations for interspecies pairwise image comparisons among images of females (a) or males (b). *n* = 205250 image pairs. **c-d)** Complements of pixel correlations. **e-f)** Pairwise centroid distances for ML embedded locations of images of females (a) or males (b). *n* = 16,734 images. **g-j**) Incongruence with genetic phylogenetic distances measured by the Euclidean distance tree similarity measure. From neighbour-joining trees based on the complement of pixel correlations for females (g) or males (h). From neighbour-joining trees based on embedding distance for females (i) or males (j). Pixel correlation shows reduced phylogenetic quality for female versus male images (median difference 0.087 3 d. p.) whereas ML embedding distance shows the same median distance to 2 decimal places for males and females. Additional analyses rescaling

each pixel correlation matrix to a proportion of its maximum confirm a higher phylogenetic distance from genetic trees for trees based on female rather than male images (median difference 0.029 to 3 d. p.). Additional analyses rescaling each pairwise embedding distance to a proportion if its maximum give median Euclidean tree distances from gene trees of 2.415 for females and 2.40 for males (to 3 d. p).

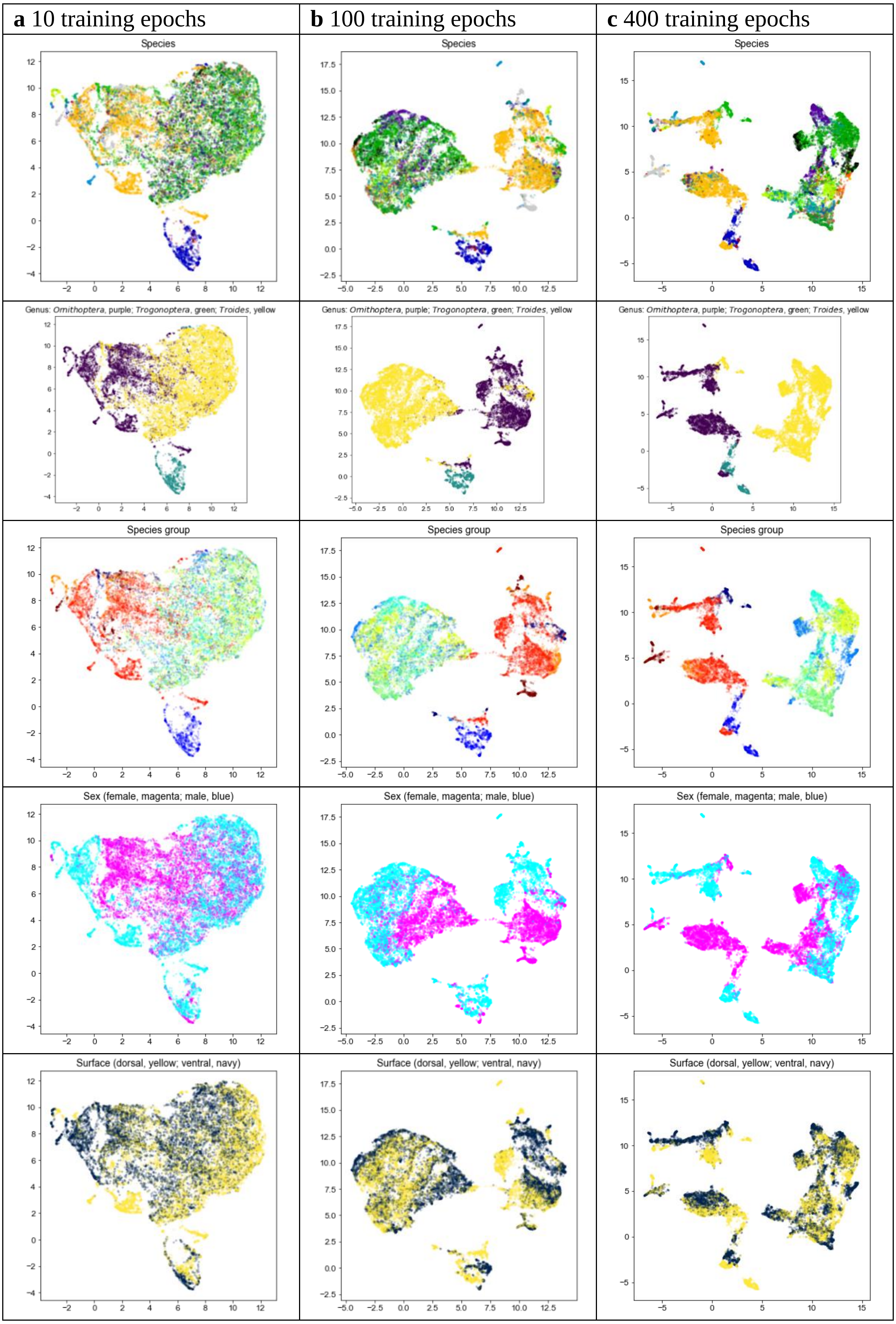
a 10 training epochs
b 100 training epochs
c 400 training epochs
Species
Genus: Ornithoptera, purple; Trogonoptera, green; Troides, yellow
Species group
Sex (female, magenta; male, blue)
Surface (dorsal, yellow; ventral, navy)

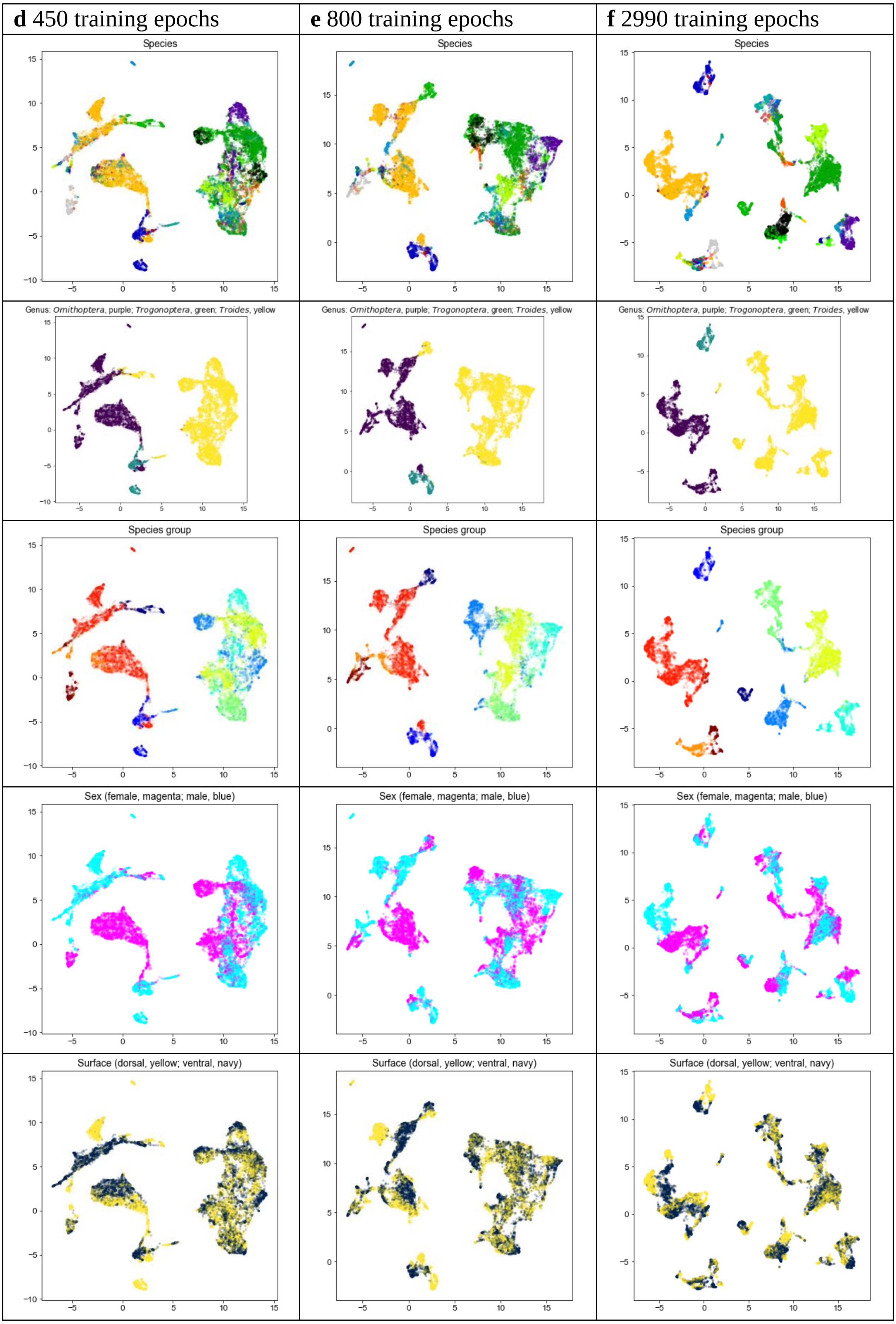
d 450 training epochs
e 800 training epochs
f 2990 training epochs
Species
Genus: Ornithoptera, purple; Trogonoptera, green; Troides, yellow
Species group
Sex (female, magenta; male, blue)
Surface (dorsal, yellow; ventral, navy)

Fig. S4. Visualisations of phenotypic embedding structure as ML training proceeds. 2D UMAP visualisations of embeddings structure at example epochs (a 10, b 100, c 400, d 450, e 1000, f, 2990) drawn from a ML training run (number 8) of 3000 total epochs. Data points represent embedding positions of 16,734 individual photographs. Colours correspond to groupings of taxonomic, phylogenetic and biological interest (top to bottom: the training label of species; genus; previously hypothesised morphological[4] and phylogenetic[28] species groups; biological sex; and imaged surface). In early training (e.g. epoch 10), the main division in 2D visualisations of embedding structure is the separation of a large phenotypic cluster containing genera *Troides* and *Ornithoptera* from a cluster primarily containing genus *Trogonoptera*, which has been found to be first diverging in independent genetic phylogenies[28]; although other structure corresponding, for example, to sex and surface is already present. Large-scale aspects of variation in the image dataset such as inter-genus and inter-sex variation are salient relatively earlier in network training (referred to as 'early' embeddings), but corresponding clusters might emerge at different specific times e.g. 10 epochs, or 20 etc. dependence on stochastic variation between independent training runs. By 400 epochs in the figured run, the three genera, for example, are all distinctly separated. At multiple points, there are interesting minor deviations from broad structural patterns, indicating for example, species from different genera with comparatively high visual similarity (e.g. epoch 100). Through early to mid-training, structure corresponding to previously hypothesised phenotypic and phylogenetic species groups is visible (e.g. epochs 100-450).

<table>
<tr><th>Tree similarity</th><th>Statistics</th></tr>
<tr><td>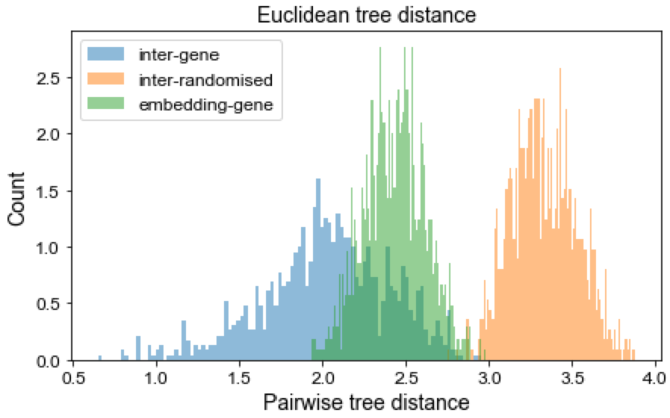
</td><td>Epochs: 10

Embedding-gene minimum=
1.935
Embedding-gene maximum=
2.981
Embedding-gene mean=
2.431
Embedding-gene median=
2.438</td></tr>
<tr><td>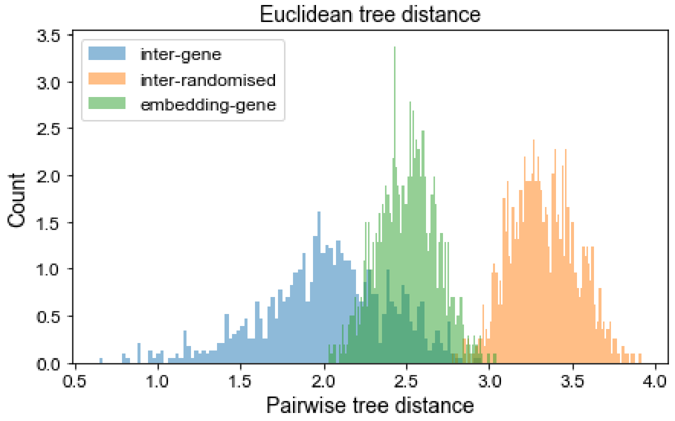
</td><td>Epochs: 100

Embedding-gene minimum=
2.032
Embedding-gene maximum=
3.039
Embedding-gene mean=
2.507
Embedding-gene median=
2.513</td></tr>
<tr><td>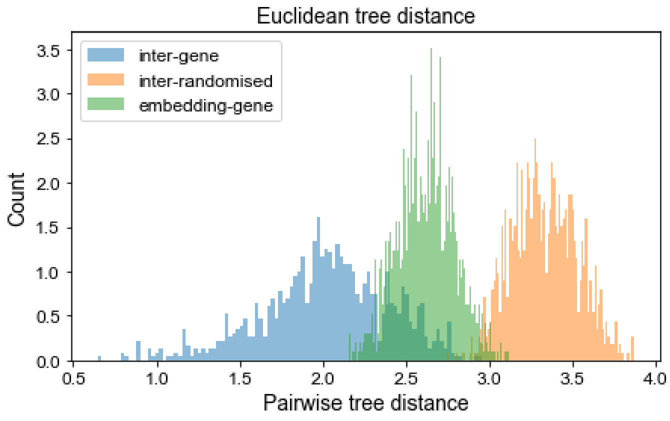
</td><td>Epochs: 800

Embedding-gene minimum=
2.153
Embedding-gene maximum=
3.120
Embedding-gene mean=
2.617
Embedding-gene median=
2.624</td></tr>
<tr><td>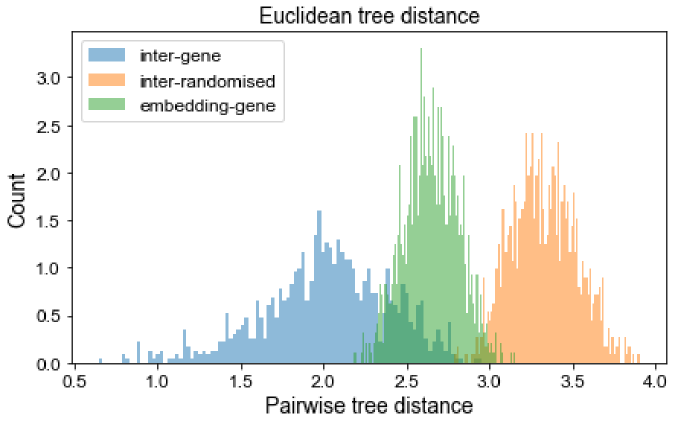
</td><td>Epochs: 2990

Embedding-gene minimum=
2.187
Embedding-gene maximum=
3.151
Embedding-gene mean=
2.650
Embedding-gene median=
2.648</td></tr>
</table>

Fig. S5. Comparison of phylogenetic trees based on ML phenotypic distance versus independent, housekeeping genes. 9900 pairwise Euclidean (branch length) tree distances: (blue) among 100 Bayesian coalescent phylogenetic trees of 30 birdwing butterfly species, based on all published DNA sequences for 4 housekeeping genes and sampled post burn-in; (orange) among 100 randomised trees; (green) between a phenotypic ML tree, reconstructed from a ML embedding of 16,734 birdwing butterfly photographs, and the genetic trees. Measures of similarity between our machine learnt phenotypic trees and independent genetic signals (green) overlap the natural range of variation in genetic signals themselves (blue), with a median and mean within this range (Inter gene-tree minimum=0.238, maximum=3.246, mean=2.073, median=2.091; further values, SI Computer Code 2), while providing unique information on visible phenotypic variation.

Tree similarity	Statistics
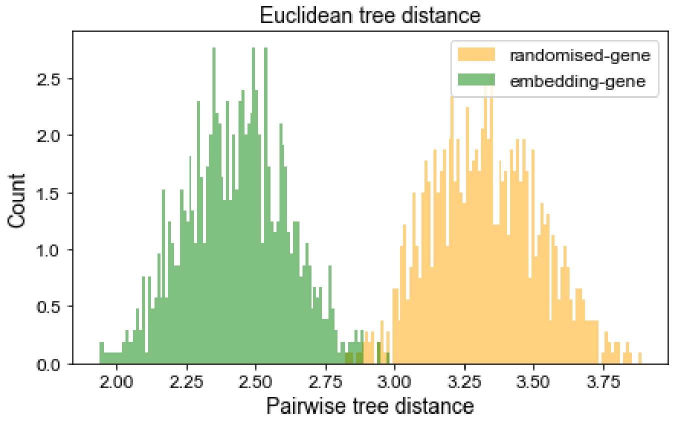	Epochs: 10<br>n = 1000<br>Omnibus normality test<br>Embedding-gene tree distances<br>statistic (s^2 + k^2) = 2.841203198513031<br>p = 0.2415686456561814<br>Randomised-gene tree distances<br>statistic (s^2 + k^2) =<br>119.68531214649178<br>p < 0.001<br>Kruskal Wallis test for equal medians<br>(null)<br>H = 2723.794195761853<br>p < 0.001<br>Mann-Whitney test for equal distributions<br>(null)<br>H = 660.0<br>p < 0.001
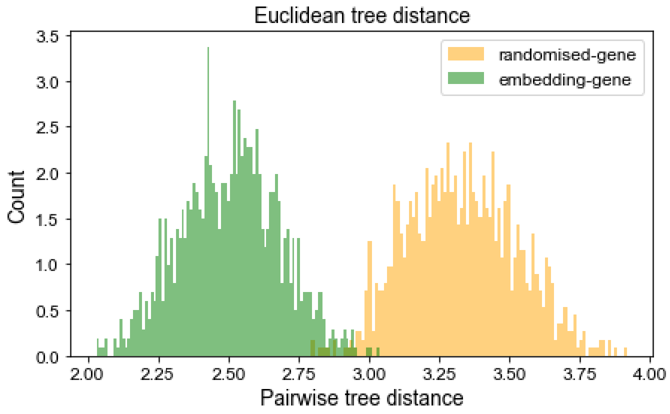	Epochs: 100<br>n = 1000<br>Omnibus normality test<br>Embedding-gene tree distances<br>statistic (s^2 + k^2) =<br>3.3710536231358077<br>p = 0.1853467634127312<br>Randomised-gene tree distances<br>statistic (s^2 + k^2) = 115.4521059900954<br>p < 0.001<br>Kruskal Wallis test for equal medians<br>(null)<br>H = 2722.2556768896975<br>p < 0.001<br>Mann-Whitney test for equal distributions<br>(null)<br>H = 2058.0<br>p < 0.001
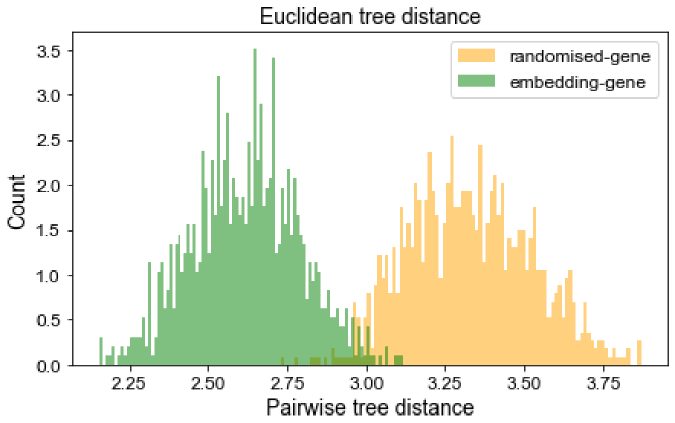	Epochs: 800<br>n = 1000<br>Omnibus normality test<br>Embedding-gene tree distances<br>statistic (s^2 + k^2) = 2.967916198080357<br>p = 0.22673845666599965<br>Randomised-gene tree distances<br>statistic (s^2 + k^2) =<br>122.43932604748193<br>p < 0.001<br>Kruskal Wallis test for equal medians<br>(null)<br>H = 2714.633333066471<br>p < 0.001<br>Mann-Whitney test for equal distributions<br>(null)<br>H = 8990.0<br>p < 0.001

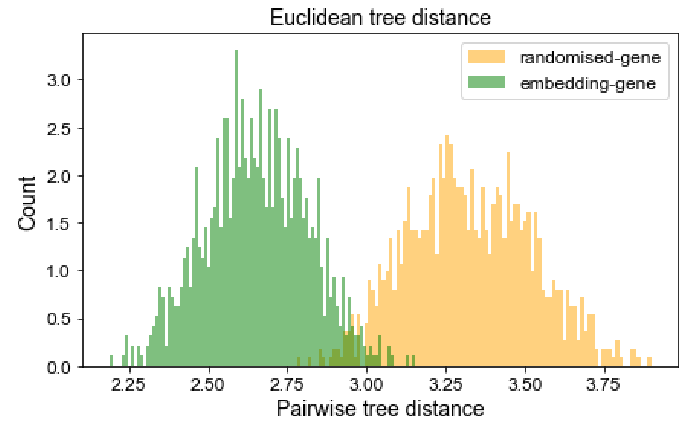

Epochs: 2990
n = 1000
Omnibus normality test
Embedding-gene tree distances
statistic (s^2 + k^2) = 4.247291176720219
p = 0.11959483926436332
Randomised-gene tree distances
statistic (s^2 + k^2) = 128.46726244195372
p < 0.001
Kruskal Wallis test for equal medians (null)
H = 2712.302143705914
p < 0.001
Mann-Whitney test for equal distributions (null)
H = 11112.0
p < 0.001

Fig. S6. Comparison to random of phylogenetic tree similarity between ML phenotypic trees versus housekeeping gene trees. Statistical comparisons of the distributions of 1000 pairwise distances between the embedding tree and a randomly selected gene tree.

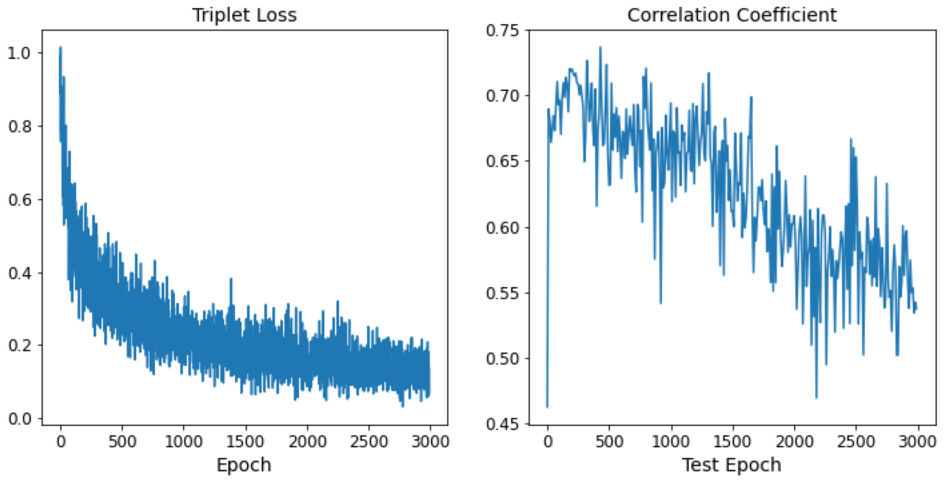

Fig. S7. Comparison of triplet loss and correlation between phenotypic and genetic distances during machine learning training. ML training run of 3000 total epochs. The triplet loss is the output of the ML optimisation function, which aims to optimally place sampled triplets of images (two with the same species label, one different) such that images of the same species are comparatively close together and triplet loss (Supplementary Computer Code 1) is minimised. The correlation coefficient is the correlation between a matrix of average distances between species in the output embedding at a given training epoch and an independent matrix of average genetic distances between species, based on 4 housekeeping genes.

**Embedding distance**	**Statistics**
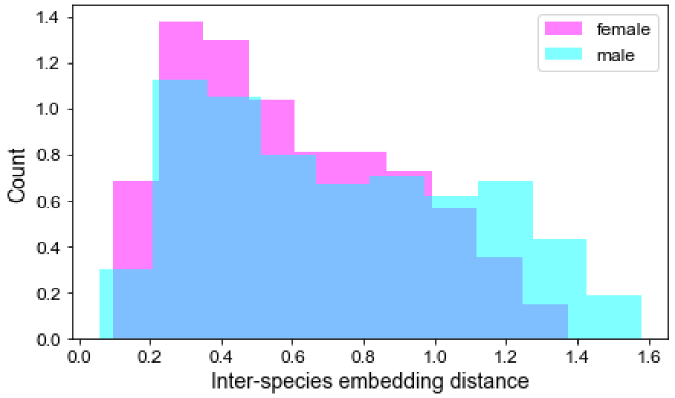	ML training run: 8<br>Epochs: 10<br>Summary statistics<br>n = 595<br>male mean = 0.7220271601282711<br>male median = 0.6715612781860143<br>male max. = 1.578657338888583<br>female mean = 0.5959420778537694<br>female median = 0.5445902329193937<br>female max. = 1.3749209999915193<br>Omnibus normality test<br>female inter-species distances<br>statistic (s^2 + k^2) = 73.85912524423745<br>p < 0.001<br>male inter-species distances<br>statistic (s^2 + k^2) =<br>171.59103014910235<br>p < 0.001<br>Statistical test results<br>Kruskal Wallis test for equal medians (null)<br>H = 29.76290350071031<br>p < 0.001<br>Mann-Whitney test for equal distributions (null)<br>H = 144674.0<br>p < 0.001
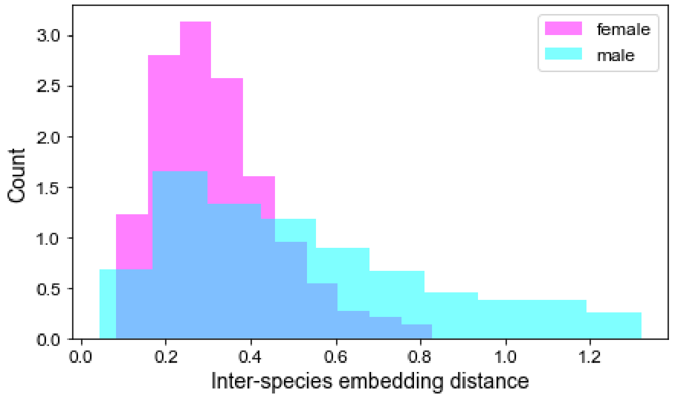	ML training run: 12<br>Epochs: 10<br>Summary statistics<br>n = 595<br>male mean = 0.5217294955515436<br>male median = 0.4549032978945065<br>male max. = 1.3197402300915475<br>female mean = 0.3209318603246412<br>female median = 0.29640723139259395<br>female max. = 0.8297412795064565<br>Omnibus normality test<br>female inter-species distances<br>statistic (s^2 + k^2) = 73.472135969773<br>p < 0.001<br>male inter-species distances<br>statistic (s^2 + k^2) = 50.44445857926421<br>p < 0.001<br>Statistical test results<br>Kruskal Wallis test for equal medians (null)<br>H = 130.33082949463642<br>p < 0.001<br>Mann-Whitney test for equal distributions (null)<br>H = 109341.0<br>p < 0.001

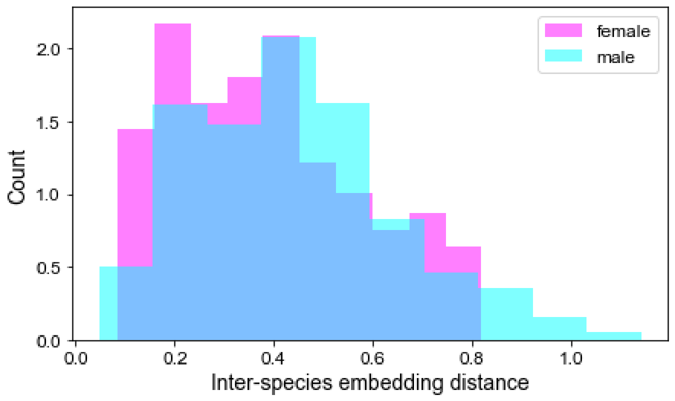	ML training run: 13<br>Epochs: 10<br>Summary statistics<br>n = 595<br>male mean = 0.4383505015403438<br>male median = 0.4193646841620437<br>male max. = 1.1408027319436038<br>female mean = 0.389996199188003<br>female median = 0.3683946492340938<br>female max. = 0.8197024770783954<br>Omnibus normality test<br>female inter-species distances<br>statistic (s^2 + k^2) = 55.56134001995156<br>p < 0.001<br>male inter-species distances<br>statistic (s^2 + k^2) = 32.84799854929418<br>p < 0.001<br>Statistical test results<br>Kruskal Wallis test for equal medians (null)<br>H = 16.013388180580023<br>p < 0.001<br>Mann-Whitney test for equal distributions (null)<br>H = 153292.0<br>p < 0.001

Fig. S8. Phenotypic distances among males versus females. Early embedding (10 epochs) distances between species centroids for males (cyan) versus females (magenta) and accompanying statistics.

**Embedding distance and sexual disparity**	**Statistics**
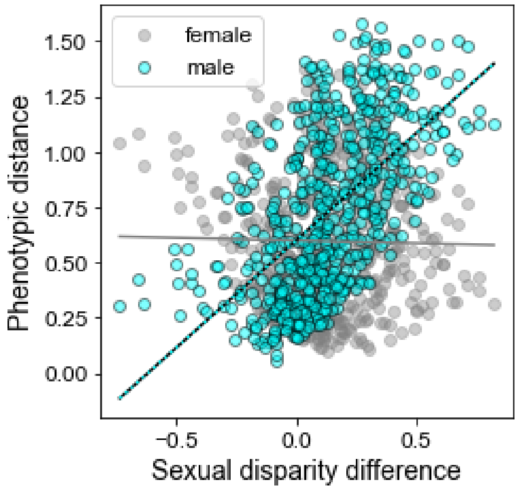	ML training run: 8<br>Epochs: 10<br>Omnibus normality test<br>Sexual disparity difference<br>statistic (s^2 + k^2) = 7.868747158228457<br>p = 0.019557946997458126<br>Female phenotypic distance<br>statistic (s^2 + k^2) = 73.85912524423745<br>$p < 0.001$<br>Male phenotypic distance<br>statistic (s^2 + k^2) = 171.59103014910235<br>$p < 0.001$<br>Statistical correlations<br>Combined<br>n=595<br>corr.=0.3849532491459061<br>$p < 0.001$<br>Males<br>n=595<br>corr.=0.6161093764331034<br>$p < 0.001$<br>Females<br>n=595<br>corr.=0.056431895515985535<br>p=0.16921737556515182
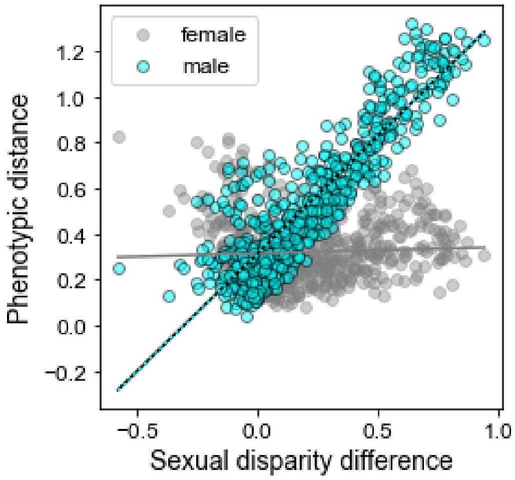	ML training run: 12<br>Epochs: 10<br>Omnibus normality test<br>Sexual disparity difference<br>statistic (s^2 + k^2) = 33.61171996797187<br>$p < 0.001$<br>Female phenotypic distance<br>statistic (s^2 + k^2) = 73.472135969773<br>$p < 0.001$<br>Male phenotypic distance<br>statistic (s^2 + k^2) = 50.44445857926421<br>$p < 0.001$<br>Statistical correlations<br>Combined<br>n=595<br>corr.=0.6066117722256689<br>$p < 0.001$<br>Males<br>n=595<br>corr.=0.8332461152042678<br>$p < 0.001$<br>Females<br>n=595<br>corr.=0.08299047094941293<br>p=0.04301234926588218

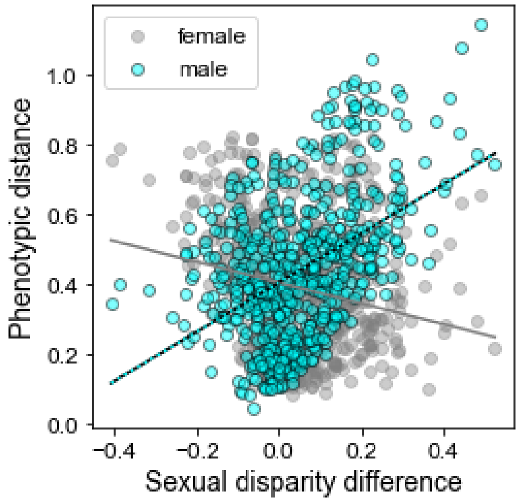	ML training run: 13<br>Epochs: 10<br>Omnibus normality test<br>Sexual disparity difference<br>statistic (s^2 + k^2) = 16.398636326924446<br>p = 0.00027484090266926127<br>Female phenotypic distance<br>statistic (s^2 + k^2) = 55.56134001995156<br>p < 0.001<br>Male phenotypic distance<br>statistic (s^2 + k^2) = 32.84799854929418<br>p < 0.001<br>Statistical correlations<br>Combined<br>n=595<br>corr.=0.09362851913187484<br>p=0.022367906332724917<br>Males<br>n=595<br>corr.=0.4023625801754504<br>p < 0.001<br>Females<br>n=595<br>corr.=-0.20590741889597003<br>p < 0.001

Fig. S9. Embedding distance and sexual disparity differences among all species. Early embeddings with emergent genus and sex structure from 3 independent ML training runs. Observed Spearman correlations between pairwise embedding distance and sexual disparity difference for males are strongly significant and positive, due to the trend in early embeddings to visually clustered females and peripheral males. In contrast, observed correlations for females are not consistently negative, with observed correlations non-significant, weakly positive or comparatively weakly negative.

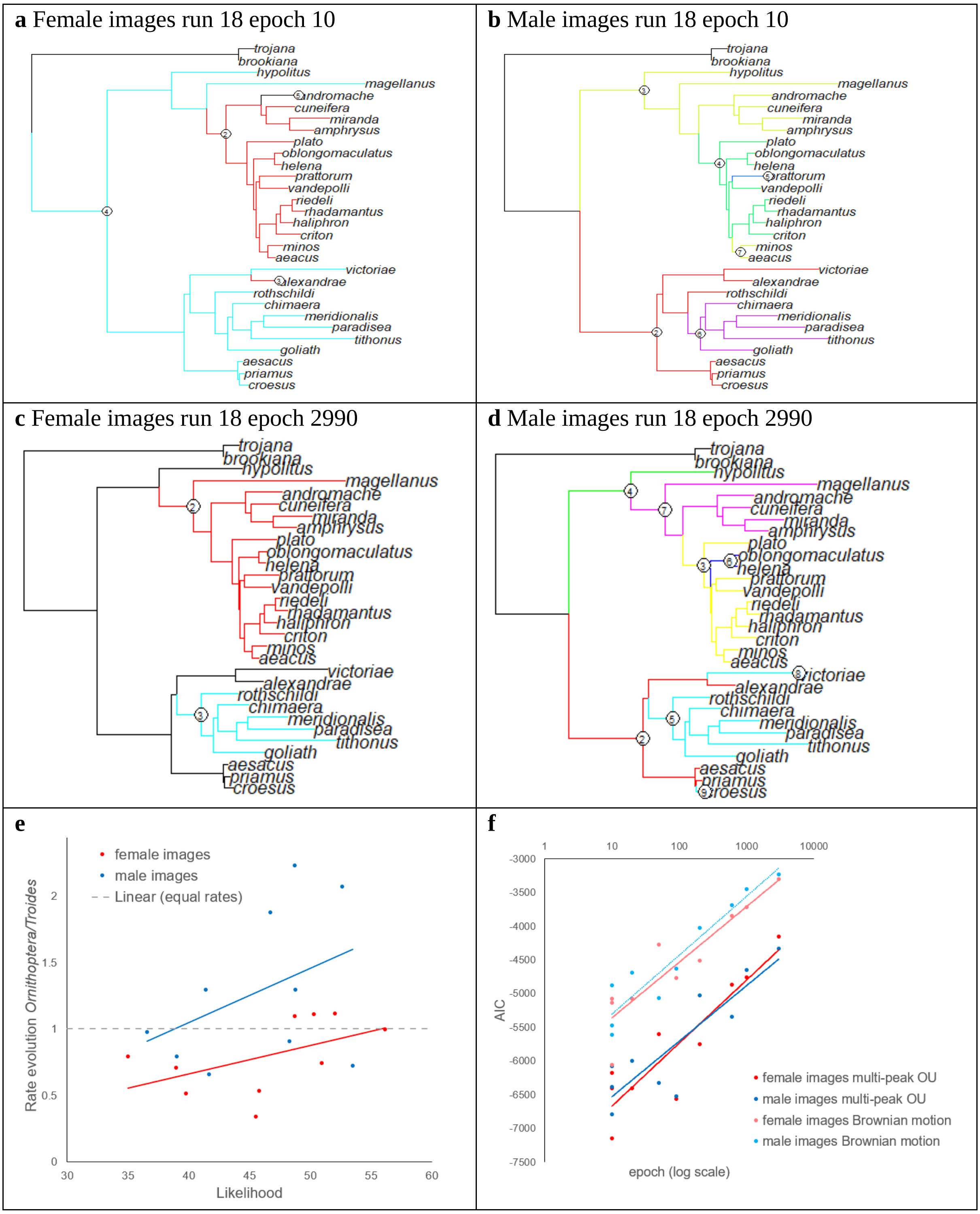

Fig. S10. a-d Multi-peak Ornstein-Uhlenbeck models fitted to 64-dimensional embedding locations for separately labelled female and male photographs of 35 species-level OTUs. Tests and statistical analyses conducted across 10 different training epochs from 3 independent ML runs (**a-f**). Four examples of best-fitting multi-peak OU regimes inferred for female (**a, c**) versus male (**b, d**) image embedding locations. Hypotheses of multiple-peak OU, single-peak OU or Brownian motion were tested against a distinct, published reference phylogeny (30 overlapping OTUs, IQ tree of ref.[29]) using the SURFACE[30] R package. In all cases a multi-peak OU model was best-fitting by the Akaike information criterion (AIC difference multi-peak minus single peak OU between -1624 and -293; multi-peak OU minus Brownian motion between -1892 and -913). The number of best-fitting selective regimes was,

in all replicates, higher for males than females (female images mean = 3.9, median = 4; male images mean = 5.8, median = 6; Shapiro Wilk normality test: females $W = 0.8325$, $p = 0.036$, males $W = 0.794$, $p = 0.123$; paired Wilcoxon test significant with $W = 55$ $p = 0.002$). Best-fitting number of convergent regimes Shapiro Wilk females $W = 0.833$, $p = 0.036$, males $W = 0.794$, $p = 0.004$; Wilcoxon $W = 12$, $p = 1$. **e, Rate of evolution in embedded image phenotypes** (inferred using the R motmot[31] package, on a reference phylogeny from ref.[29] **a-d**) for clade *Ornithoptera* divided by that for clade *Troides* (data points show average value across 64 embedding axes for each of the 10 repeated embedding samples). Shapiro Wilk female images $W = 0.9155$, $p = 0.402$; males $W = 0.879$ $p = 0.142$; paired *t*-test $t = -3.232$, $p = 0.018$; mean females = 0.794, males = 1.283. **f, Fit of a comparative model on the reference phylogeny given ML training epoch.** Female and male multi-peak OU, single-peak OU and Brownian motion AIC values, Shapiro Wilk $p > 0.246$; linear regression against log10 transformed epoch $r^2 > 0.81$, $p < 0.0008$.

Supplementary References

1. Wilts, B. D., Matsushita, A., Arikawa, K. & Stavenga, D. G. Spectrally tuned structural and pigmentary coloration of birdwing butterfly wing scales. *J. R. Soc. Interface* **12**, 20150717 (2015).
2. Kazama, M. *et al.* Species-dependent microarchitectural traits of iridescent scales in the triad taxa of *Ornithoptera* birdwing butterflies. *Entomol. Sci.* **20**, 255–269 (2017).
3. Vigneron, J. P. *et al.* Correlated diffraction and fluorescence in the backscattering iridescence of the male butterfly *Troides magellanus* (Papilionidae). *Phys. Rev. E* **78**, 021903 (2008).
4. Haugum, J. & Low, A. M. *A Monograph of the Birdwing Butterflies*. (Scandinavian Science Press, 1978).
5. Matsuka, H. *Natural History of Birdwing Butterflies*. (Matsuka Shuppan, Tokyo, Japan, 2001).
6. Straatman, R. Notes on the biology and hostplant associations of *Ornithoptera priamus urvilleanus* and *O. victoriae* (Papilionidae). *J. Lepidopterists Soc.* **23**, 69–76 (1969).

7. Wallace, A. R. *The Malay Archipelago: The Land of the Orang-Utan and the Bird of Paradise; a Narrative of Travel, with Studies of Man and Nature*. (Harper & Brothers, 1869).
8. D'Abrera, B. *Birdwing Butterflies of the World*. (Hamlyn Publishing Group Limited, London, 1976).
9. Tutt, J. W. Notes on the behaviour of Rajah Brooke's birdwing butterfly, *Trogonoptera brookiana brookiana*. *Entomol. Rec. J. Var.* **92**, 98–101 (1980).
10. Parsons, M. Notes on the courtship of *Troides oblongomaculatus papuensis* (Papilionidae) in Papua New Guinea. *J. Lepidopterists Soc.* (1983).
11. Priamus *Birdwing Butterfly Courtship*. (2013).
12. *Mating Dance Cairns Birdwing Butterfly*. (2021).
13. *Birdwing Butterflies Male & Female SloMo*. (2021).
14. Clutton-Brock, T. We do not need a Sexual Selection 2.0—nor a theory of Genial Selection. *Anim. Behav.* **79**, e7–e10 (2010).
15. Rosvall, K. A. Intrasexual competition in females: evidence for sexual selection? *Behav. Ecol.* **22**, 1131–1140 (2011).
16. Darwin, C. *The Descent of Man, and Selection in Relation to Sex.* (D. Appleton and company, New York, 1871).
17. Cooper, I. A., Brown, J. M. & Getty, T. A role for ecology in the evolution of colour variation and sexual dimorphism in Hawaiian damselflies. *J. Evol. Biol.* **29**, 418–427 (2016).
18. Kunte, K. Mimetic butterflies support Wallace's model of sexual dimorphism. *Proc. R. Soc. B Biol. Sci.* **275**, 1617–1624 (2008).
19. Vollrath, F. Dwarf males. *Trends Ecol. Evol.* **13**, 159–163 (1998).

20. Wallace, A. R. *Darwinism: An Exposition of the Theory of Natural Selection with Some of Its Applications.* (Macmillan & Co., London & New York, 1889).

21. Ohsaki, N. A common mechanism explaining the evolution of female-limited and both-sex Batesian mimicry in butterflies. *J. Anim. Ecol.* **74**, 728–734 (2005).

22. Parsons, M. J. Gondwanan evolution of the troidine swallowtails (Lepidoptera: Papilionidae): cladistic reappraisals using mainly immature stage characters, with focus on the birdwings *Ornithoptera* Boisduval. *Bull. Kitakyushu Mus. Nat. Hist.* **15**, 43–118 (1996).

23. Bateman, A. J. Intra-sexual selection in *Drosophila*. *Heredity* **2**, 349–368 (1948).

24. Jones, A. G., Ratterman, N. L. & Paczolt, K. A. The adaptive landscape in sexual selection research. *Adapt. Landsc. Evol. Biol.* 110–122 (2012).

25. Westerman, E. L. *et al.* Does male preference play a role in maintaining female limited polymorphism in a Batesian mimetic butterfly? *Behav. Processes* **150**, 47–58 (2018).

26. Stewart, A. D. & Rice, W. R. Arrest of sex-specific adaptation during the evolution of sexual dimorphism in *Drosophila*. *Nat. Ecol. Evol.* **2**, 1507–1513 (2018).

27. MacLeod, N. & Kolska Horwitz, L. Machine-learning strategies for testing patterns of morphological variation in small samples: sexual dimorphism in gray wolf (*Canis lupus*) crania. *BMC Biol.* **18**, 113 (2020).

28. Condamine, F. L. *et al.* Deciphering the evolution of birdwing butterflies 150 years after Alfred Russel Wallace. *Sci. Rep.* **5**, 1–11 (2015).

29. Allio, R. *et al.* Genome-wide macroevolutionary signatures of key innovations in butterflies colonizing new host plants. *Nat. Commun.* **12**, 354 (2021).

30. Ingram, T. & Mahler, D. L. SURFACE: detecting convergent evolution from comparative data by fitting Ornstein-Uhlenbeck models with stepwise Akaike Information Criterion. *Methods Ecol. Evol.* **4**, 416–425 (2013).

31. Thomas, G. H. & Freckleton, R. P. MOTMOT: models of trait macroevolution on trees. *Methods Ecol. Evol.* **3**, 145–151 (2012).
