# Supplementary material for: Male and female contributions to diversity among birdwing butterfly images
Source: Commun Biol. 2024 Jul 1;7:774. doi: 10.1038/s42003-024-06376-2 (PMC11217504; doi:10.1038/s42003-024-06376-2)
Supplement: Supplementary file 2 — Description of Additional Supplementary Files [file 42003_2024_6376_MOESM2_ESM.pdf]

Description of Additional Supplementary Files

File name: Supplementary Data 1

Description: Image label data.

File name: Supplementary Data 2

Description: Accession numbers of publicly available genetic data used in this study.

File name: Supplementary Data 3

Description: Processed photographic data used in machine learning and additional analyses.

File name: Supplementary Data 4

Description: Taxonomic labels formatted for machine learning analyses.

File name: Supplementary Data 5

Description: Representative trained machine learning model.

File name: Supplementary Data 6

Description: Matrix of genetic distances for comparisons in machine learning.

File name: Supplementary Data 7-10

Description: Source data for figures 1-4, respectively

File name: Supplementary Software 1

Description: Machine learning computer code in Python ipnyb format

File name: Supplementary Software 2

Description: BEAST input file for genetic phylogenetic analyses in xml format

File name: Supplementary Software 3

Description: Comparative pixel analysis computer code in Python ipnyb format
