# Supplementary material for: Male and female contributions to diversity among birdwing butterfly images
Source: Commun Biol. 2024 Jul 1;7:774. doi: 10.1038/s42003-024-06376-2 (PMC11217504; doi:10.1038/s42003-024-06376-2)
Supplement: Supplementary file 11 — Reporting Summary [file 42003_2024_6376_MOESM11_ESM.pdf]

nature portfolio

Corresponding author(s): J. Hoyal Cuthill

Last updated by author(s): Apr 22, 2024

Reporting Summary

Nature Portfolio wishes to improve the reproducibility of the work that we publish. This form provides structure for consistency and transparency in reporting. For further information on Nature Portfolio policies, see our Editorial Policies and the Editorial Policy Checklist.

Statistics

For all statistical analyses, confirm that the following items are present in the figure legend, table legend, main text, or Methods section.

n/a	Confirmed	
☐	☒	The exact sample size (*n*) for each experimental group/condition, given as a discrete number and unit of measurement
☐	☒	A statement on whether measurements were taken from distinct samples or whether the same sample was measured repeatedly
☐	☒	The statistical test(s) used AND whether they are one- or two-sided<br>*Only common tests should be described solely by name; describe more complex techniques in the Methods section.*
☒	☐	A description of all covariates tested
☐	☒	A description of any assumptions or corrections, such as tests of normality and adjustment for multiple comparisons
☒	☐	A full description of the statistical parameters including central tendency (e.g. means) or other basic estimates (e.g. regression coefficient) AND variation (e.g. standard deviation) or associated estimates of uncertainty (e.g. confidence intervals)
☐	☒	For null hypothesis testing, the test statistic (e.g. F, t, r) with confidence intervals, effect sizes, degrees of freedom and P value noted<br>*Give P values as exact values whenever suitable.*
☒	☐	For Bayesian analysis, information on the choice of priors and Markov chain Monte Carlo settings
☒	☐	For hierarchical and complex designs, identification of the appropriate level for tests and full reporting of outcomes
☐	☒	Estimates of effect sizes (e.g. Cohen's d, Pearson's r), indicating how they were calculated

Our web collection on statistics for biologists contains articles on many of the points above.

Software and code

Policy information about availability of computer code

Data collection	No software was used to collect the primary digital photographic data which is publicly available via the NHM data portal or publicly accessioned sequence DNA
Data analysis	Custom software was written for data analysis and is provided with the submission as supplementary computer code, including machine learning software ButterflyNet version 1.2

For manuscripts utilizing custom algorithms or software that are central to the research but not yet described in published literature, software must be made available to editors and reviewers. We strongly encourage code deposition in a community repository (e.g. GitHub). See the Nature Portfolio guidelines for submitting code & software for further information.

Data

Policy information about availability of data

All manuscripts must include a data availability statement. This statement should provide the following information, where applicable:

- Accession codes, unique identifiers, or web links for publicly available datasets
- A description of any restrictions on data availability
- For clinical datasets or third party data, please ensure that the statement adheres to our policy

Data Availability Statement
All photographic data used in this study and metadata are publicly available at https://data.nhm.ac.uk/dataset/collection-specimens.

Processed photographic data used in the analyses and their taxonomic label data are also provided in the SI
.
Accession numbers of publicly available genetic data, used in this study are provided as SI Table 2.
Computer code used to perform machine learning analyses, and trained ML models, are provided as Supplementary Computer Code 1 (ButterflyNet Version 1.2).
Aligned genetic data, and accompanying analytical parameters, are provided as a BEAST xml input file in Supplementary Computer Code 2. Computer code for pixel correlation analyses are provided as Supplementary Computer Code 3.

Research involving human participants, their data, or biological material

Policy information about studies with human participants or human data. See also policy information about sex, gender (identity/presentation), and sexual orientation and race, ethnicity and racism.

Reporting on sex and gender	NA: no human participants
Reporting on race, ethnicity, or other socially relevant groupings	NA: no human participants
Population characteristics	NA: no human participants
Recruitment	NA: no human participants
Ethics oversight	NA: no human participants

Note that full information on the approval of the study protocol must also be provided in the manuscript.

Field-specific reporting

Please select the one below that is the best fit for your research. If you are not sure, read the appropriate sections before making your selection.

☐ Life sciences ☐ Behavioural & social sciences ☒ Ecological, evolutionary & environmental sciences

For a reference copy of the document with all sections, see nature.com/documents/nr-reporting-summary-flat.pdf

Ecological, evolutionary & environmental sciences study design

All studies must disclose on these points even when the disclosure is negative.

Study description	Machine learning (spatial embedding) analysis of 16,734 dorsal and ventral photographs of historical specimens of birdwing butterflies (with raw photographic data sourced from the Natural History Museum, NHMUK public digital data repository)
Research sample	16,734 dorsal and ventral photographs of historical specimens of birdwing butterflies (with raw photographic data sourced from the NHMUK public digital data repository)
Sampling strategy	Exhaustive sampling of all available NHMUK photographic dataset for the study group of birdwing butterflies
Data collection	Published photographic data available from the NHMUK data portal
Timing and spatial scale	NA
Data exclusions	Some photographs not meeting data quality benchmarks were excluded as described in the methods section of the paper
Reproducibility	Computer code required to replicate analyses is provided as supplementary material
Randomization	Group allocation to species and sex is biologically determined and recorded in the NHMUK data archives. Relevant museum curation and data processing protocols are described in the methods section of the paper.
Blinding	Not applicable as group allocation to species and sex is biologically determined and recorded in the NHMUK data archives.

Did the study involve field work? ☐ Yes ☒ No

Reporting for specific materials, systems and methods

We require information from authors about some types of materials, experimental systems and methods used in many studies. Here, indicate whether each material, system or method listed is relevant to your study. If you are not sure if a list item applies to your research, read the appropriate section before selecting a response.

Materials & experimental systems

n/a	Involved in the study
☒	☐ Antibodies
☒	☐ Eukaryotic cell lines
☒	☐ Palaeontology and archaeology
☒	☐ Animals and other organisms
☒	☐ Clinical data
☒	☐ Dual use research of concern
☒	☐ Plants

Methods

n/a	Involved in the study
☒	☐ ChIP-seq
☒	☐ Flow cytometry
☒	☐ MRI-based neuroimaging

Plants

Seed stocks	NA: no plants used
Novel plant genotypes	NA: no plants used
Authentication	NA: no plants used
